# Lactate metabolism and protein lactylation in inflammatory and tumor microenvironments

**DOI:** 10.1186/s43556-026-00558-6

**Published:** 2026-08-27

**Authors:** Huaqing Lei, Yulin Bai, Jiaqi Tang, Xiaoyan Pu, Jiarui Lan, Xiangyu Lei, Dan Cai, Shuang Gou, Lin Liu, Siyu Hao, Yu Chen, Yueshui Zhao, Jing Shen, Xu Wu, Mingxing Li, Meijuan Chen, Xiaobing Li, Yuhong Sun, Li Gu, Wanping Li, Zhangang Xiao, Yan Li, Yan Zhang, Hanyu Li, Fukuan Du

**Affiliations:** 1 Laboratory of Molecular Pharmacology, Department of Pharmacology, School of Pharmacy, Southwest Medical University, Luzhou, Sichuan 646000 China; 2 South Sichuan Institute of Translational Medicine, Luzhou, Sichuan 646000 China; 3 Cell Therapy & Cell Drugs of Luzhou Key Laboratory, Luzhou, Sichuan 646000 China; 4 Department of Pharmacy, Chongqing Medical University, Chongqing, 400016 China; 5 Department of Oncology, Luzhou People’s Hospital, Luzhou, Sichuan 646000 China; 6 Guang’an People’s Hospital, Guang’an, Sichuan 638000 China

**Keywords:** Lactate metabolism, Protein lactylation, Histone lactylation, Non-histone lactylation, Tumor microenvironment, Inflammatory microenvironment

## Abstract

Lactate and lactate-mediated protein lactylation are no longer viewed merely as accompanying phenomena of enhanced glycolysis, hypoxic responses, or tissue acidification. They are now recognized as an important regulatory axis that links local metabolic stress to chromatin regulation and altered protein function. With the rapid development of research on histone and non-histone lactylation, lactate-related signals have been implicated in inflammatory injury and repair, fibrotic remodeling, tumor immune escape, and therapy resistance. However, current studies often conflate elevated lactate levels, global increases in lactylation, site-specific lactylation events, and disease-dependent functional consequences, which can lead to overinterpretation of both the biological impact and therapeutic value of lactylation. This review first summarizes lactate production, transport, and local homeostatic regulation, then discusses the biochemical basis and detection strategies of protein lactylation. It further examines how histone lactylation reshapes transcriptional programs and how non-histone lactylation influences immune regulation by altering protein fate and signaling execution. Considering the distinct features of inflammatory and tumor microenvironments, this review compares the functional outputs of the lactate–lactylation axis during stage-specific inflammatory responses and persistent tumor-associated stress, with particular emphasis on its translational significance in immune checkpoint regulation, impaired antigen presentation, and therapeutic resistance. We propose a stratified framework for interpreting lactate-related events, distinguishing metabolic stress readouts, functional regulatory events, and disease-dependent nodes. This framework may support patient stratification, lesion-selective delivery, dynamic monitoring, and the design of precise combination therapies.

## Introduction

Lactate was historically viewed primarily as a glycolytic end product associated with impaired oxidative metabolism [1]. This view has been substantially revised by isotope-tracing studies and spatially resolved multi-omics, which have shown that lactate can serve as a carbon source for the tricarboxylic acid cycle, support oxidative metabolism, and exhibit pronounced cell-type and regional heterogeneity [2–4]. Beyond its role as a metabolic substrate, lactate can be exchanged between cells through proton-coupled monocarboxylate transport and can act through receptor-mediated sensing to influence local tissue responses [5–7]. Because tissue lactate abundance reflects the integrated balance among production, transport, cellular uptake, reutilization, and clearance, local accumulation is better interpreted as a disruption of lactate homeostasis within a defined cellular and spatial context rather than as a uniform marker of hypoxia or glycolytic activity [4, 8]. Accordingly, contemporary lactate research increasingly focuses on cellular sources, spatial distribution, metabolic flux, and functional consequences.

The discovery of protein lactylation has further expanded the conceptual framework of lactate biology [9]. As a lysine acylation process coupled to lactate availability and cellular metabolic state, protein lactylation provides a molecular route through which lactate-associated metabolic changes can be translated into chromatin regulation and direct control of protein function. Histone lactylation primarily shapes site- and locus-dependent chromatin states and transcriptional programs, whereas non-histone lactylation can regulate protein activity, stability, intracellular trafficking, subcellular organization, and molecular interactions. These regulatory outputs have been implicated in inflammatory injury and resolution, tissue repair and fibrotic remodeling, tumor immune escape, and therapy resistance [9–11]. However, increases in lactate abundance or global lactylation alone do not establish a causal lactylation-dependent mechanism, and should be distinguished from site-specific functional events and therapeutically actionable dependencies.

Inflammatory microenvironments and the tumor microenvironment (TME) provide two representative pathological contexts for understanding the lactate–lactylation axis. In inflammatory microenvironments, lactate exposure often changes dynamically with immune activation, tissue injury, and repair processes, and its functions are consequently stage-dependent and plastic [12, 13]. In the TME, lactate enrichment is usually more sustained and spatially organized, and it acts together with hypoxic adaptation, stromal remodeling, immunosuppression, and therapeutic resistance to create lesion-dependent stress [14]. These differences underscore the need for a context-dependent interpretation of the lactate–lactylation axis.

Accordingly, this review first summarizes lactate production, transport, reutilization, and local homeostatic regulation, and then examines the biochemical formation, detection, and evidence-based interpretation of protein lactylation. It next compares histone and non-histone lactylation and discusses their context-dependent and complementary roles in inflammatory microenvironments and the TME. Finally, it examines emerging therapeutic strategies targeting lactate production, transport, sensing, lactylation-regulatory machinery, and functional lactylation events, with particular attention to combination therapy, patient stratification, lesion-selective delivery, and dynamic monitoring. Throughout this review, lactate-related findings are organized into metabolic stress readouts, functional regulatory events, and disease-dependent nodes, providing a framework for distinguishing descriptive associations from stronger mechanistic and translational evidence.

## Lactate production, transport, and local homeostasis

Lactate is a major product of glycolysis and can also actively regulate mitochondrial oxidative metabolism [15]. At physiological pH, lactate exists predominantly as the lactate anion, although biomedical literature commonly refers to it collectively as “lactate.” Current evidence indicates that lactate participates in energy metabolism, intercellular metabolic exchange, and microenvironmental remodeling [16, 17]. Elevated tissue lactate therefore reflects a shift in the balance among production, transport, cellular reutilization, and local or systemic clearance [8]. This section summarizes the basis of lactate stress from three perspectives: lactate production, transmembrane transport, and local homeostatic regulation.

### Lactate production and cellular sources

**Fig. 1:**
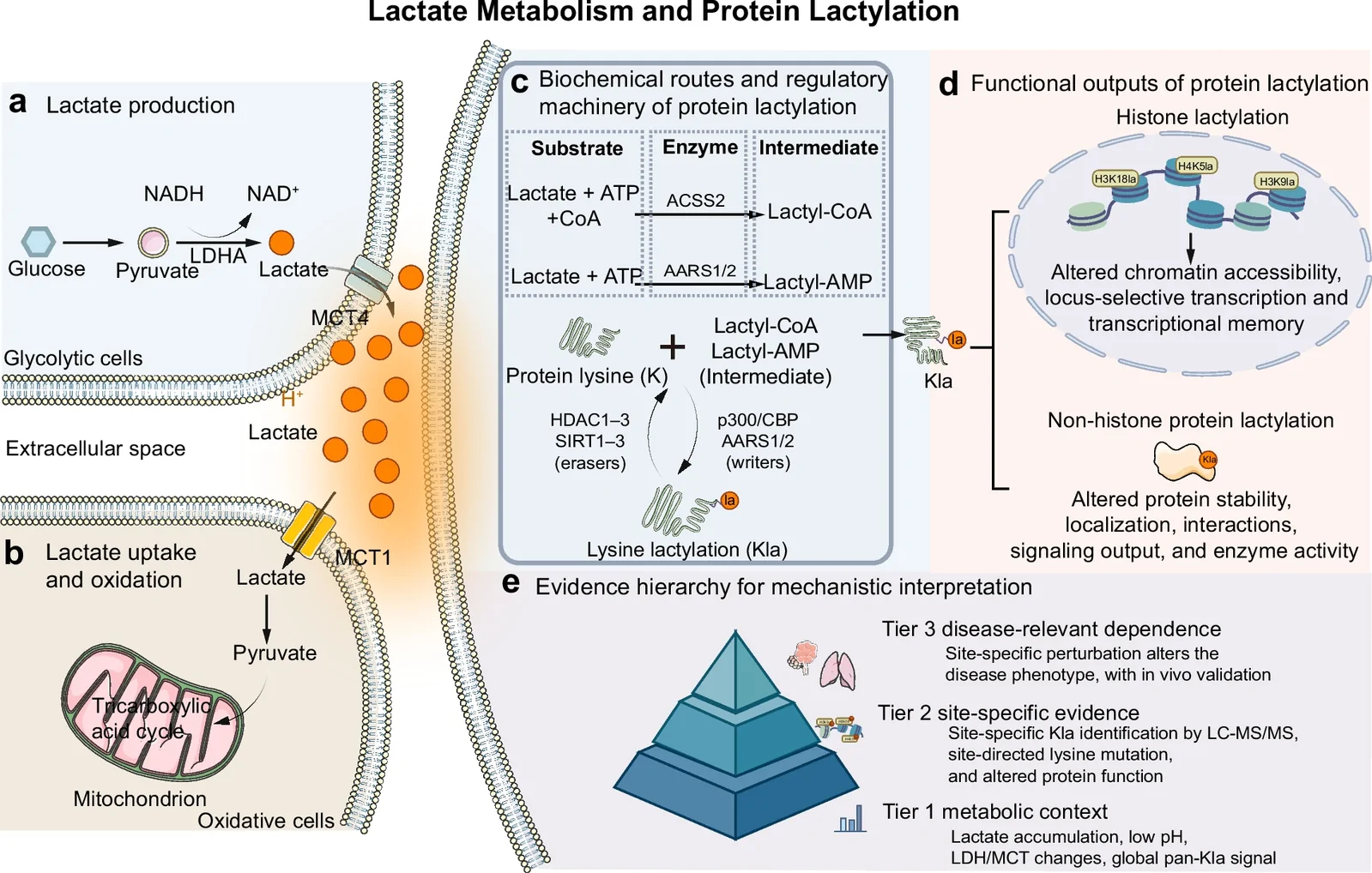
Foundational overview of lactate metabolism, protein lactylation, and mechanistic evidence. This schematic summarizes lactate production and exchange, the biochemical routes and regulatory machinery of protein lactylation, its functional outputs, and the hierarchy of mechanistic evidence. **a**, **b** Glycolytic cells produce and export lactate through MCT4, whereas oxidative cells import lactate through MCT1 for mitochondrial oxidation. **c** ACSS2- and AARS1/2-associated routes generate lactyl-CoA and lactyl-AMP, respectively, and reported writers and erasers regulate protein lysine lactylation. **d** Histone and non-histone lactylation regulate chromatin states and protein function, respectively. **e** Evidence progresses from metabolic context to site-specific functional validation and disease-relevant dependence. Orange dots represent lactate; orange “Kla” symbols and the suffix “la” denote lysine lactylation at the indicated residue

Lactate is generated primarily through the reduction of pyruvate. After glucose is converted to pyruvate through glycolysis, pyruvate is further reduced to lactate by lactate dehydrogenase, accompanied by the oxidation of NADH to NAD⁺, thereby sustaining glycolytic flux (Fig. 1a). Because lactate dehydrogenase (LDH) catalyzes a reversible reaction, the interconversion between lactate and pyruvate is jointly shaped by the NADH/NAD⁺ state, substrate availability, and cellular metabolic demand [18]. Conditions such as hypoxia [19], rapid proliferation [20], inflammatory activation [21], and tissue injury [22] can all increase glycolytic flux and promote lactate production. Thus, lactate production is shaped not only by oxygen availability, but also by cytosolic redox balance and the proliferative or activation state of the cell.

Local lactate burden arises from multiple cellular sources. Activated immune cells can rapidly increase glycolysis and release lactate during inflammatory responses [23–25], whereas hypoxia and tissue injury can promote lactate production through constrained oxidative metabolism or glycolytic reprogramming [22, 26]. Fibroblasts and other stromal cells can also contribute to lactate accumulation during tissue remodeling and pathological stress [27, 28]. In tumors, highly glycolytic cancer cells often represent a major source of lactate production [29, 30], but metabolic exchange involving immune cells [31, 32], endothelial cells [33, 34], and stromal cells [35] also shapes the local distribution of lactate. Lactate should therefore be understood not only as an intracellular metabolite but also as a tissue microenvironmental variable whose biological meaning depends on the relationship among lactate-producing cells, exposed regions, and responding cell populations.

### Monocarboxylate transporter-mediated lactate transport and intercellular exchange

Transmembrane lactate transport is mediated mainly by monocarboxylate transporters, among which monocarboxylate transporter 1 (MCT1) and monocarboxylate transporter 4 (MCT4) are the best-characterized members [36, 37]. MCTs transport monocarboxylates through proton-coupled mechanisms; therefore, lactate flux is often accompanied by H⁺ transport and can influence extracellular pH, tissue acidification, and the local microenvironmental state [36, 37]. Highly glycolytic cells typically export lactate through MCT4 to sustain glycolytic flux and maintain acid–base balance, whereas cells with stronger oxidative metabolic capacity can import lactate through MCT1 and convert it to pyruvate for entry into the tricarboxylic acid cycle or other metabolic pathways (Fig. 1a,b) [38–40].

The net direction of MCT1- and MCT4-mediated lactate transport is determined primarily by transmembrane lactate and proton gradients and is therefore influenced by intra- and extracellular pH and the metabolic state that establishes these gradients [36, 37]. This reversibility allows some cells to relieve reductive pressure by releasing lactate, while others use lactate as an oxidative substrate or input for downstream regulatory programs, thereby establishing local metabolic coupling [41, 42]. Accordingly, lactate transport helps determine not only whether lactate accumulates locally, but also how it is redistributed among neighboring cell populations within tissue microenvironments.

### Local homeostasis and spatial heterogeneity of lactate exposure

Under physiological conditions, lactate production, transport, reutilization, and clearance are maintained in dynamic balance [8]. Lactate can be reused by neighboring cells or enter the circulation for further metabolism by the liver, kidneys, heart, skeletal muscle, and other tissues [16, 43]. When enhanced glycolysis, restricted tissue perfusion, insufficient oxygen supply, and reduced systemic clearance collectively disrupt this balance, lactate can accumulate in local tissues [8, 44]. Such accumulation is usually not spatially uniform, but is shaped by vascular oxygen delivery, tissue architecture, cellular composition, and local metabolic state [4, 44].

In complex microenvironments such as inflammatory lesions and tumors, lactate exposure can show pronounced spatial and temporal heterogeneity [45–48]. In inflammatory sites, lactate levels may fluctuate with immune cell activation [23–25] and across tissue injury and repair [49]. In tumors, lactate enrichment often overlaps with hypoxic regions [50], abnormal vasculature, and stromal remodeling [4, 33, 51]. Depending on the responding cell type and local metabolic context, lactate may be reutilized as a metabolic substrate [51], sensed through G protein-coupled receptor 81 (GPR81) [52], or coupled to protein lactylation [53]. Therefore, disrupted lactate homeostasis primarily defines a background of metabolic stress, whereas its functional significance should be interpreted according to the responding cell type, the mode of lactate utilization or sensing, and, where lactylation is implicated, the modified substrate or site and supporting perturbation evidence.

## Protein lactylation and evidence-based functional interpretation

Protein lactylation extends lactate research from metabolic stress and microenvironmental acidification to post-translational regulation. Lactate can serve as a source of lactyl groups and participate in chromatin regulation and protein functional control through lysine lactylation (Kla) [9, 11, 53, 54]. This modification links lactate-associated metabolic states to protein regulatory networks, providing a molecular route through which metabolic changes can be translated into transcriptional and protein-functional outputs [55]. Accordingly, disrupted lactate homeostasis primarily defines the metabolic context, whereas protein lactylation shifts the focus toward evaluating causal relationships among specific modification sites, protein behavior, and pathological phenotypes.

### Lactate metabolism links lactate exposure to protein regulation

Protein lactylation first attracted broad attention with the discovery of histone lysine lactylation. In bacterially challenged M1 macrophages, enhanced glycolysis and lactate accumulation were accompanied by increased histone lactylation. During the late phase of M1 macrophage polarization, histone lactylation was associated with the expression of homeostatic and repair-related genes such as Arg1, suggesting that lactate-linked metabolic states can be converted into defined transcriptional programs through chromatin modification [9]. This finding extended lactate biology beyond metabolic regulation into epigenetic and transcriptional control.

**Fig. 2:**
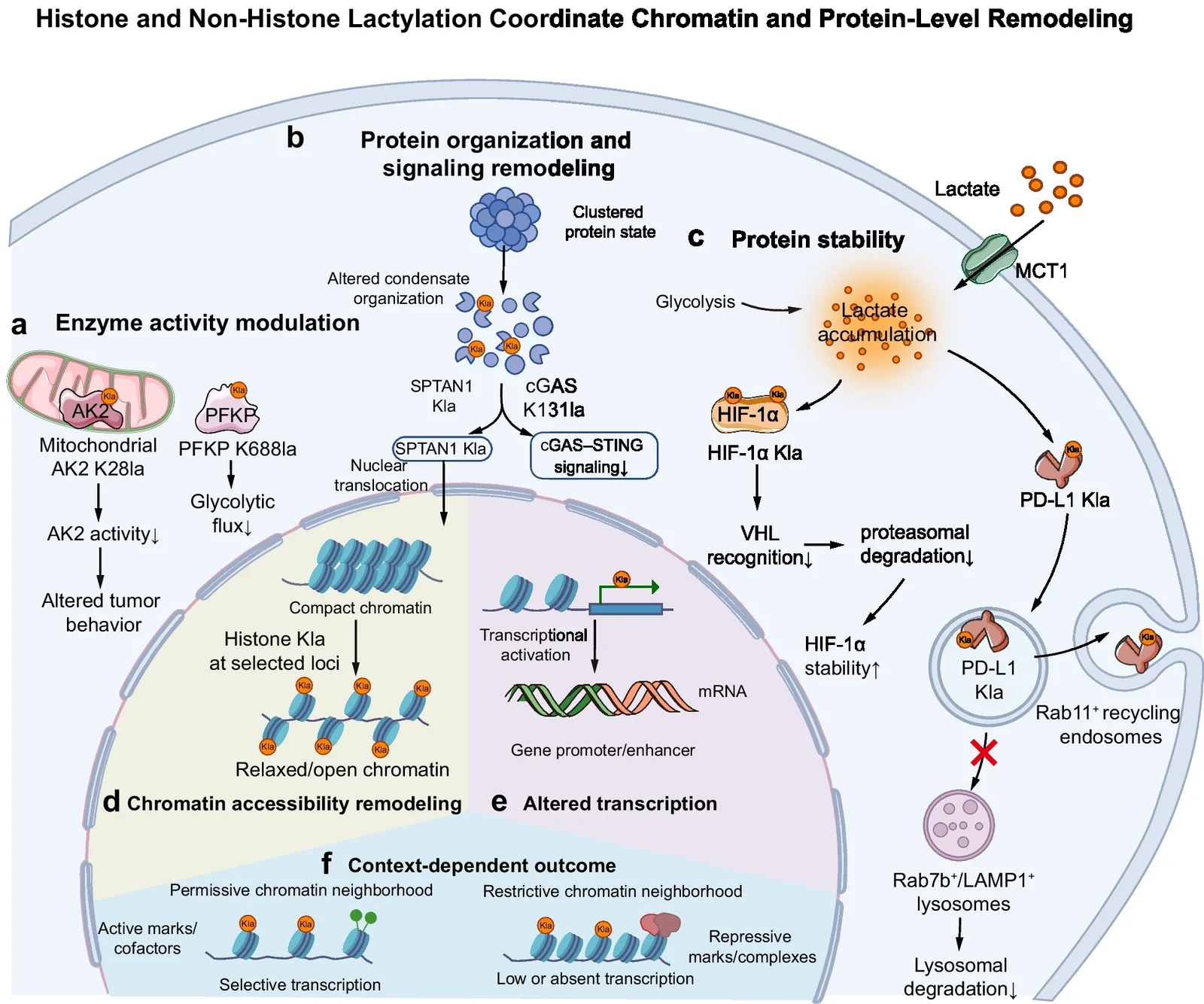
Histone and non-histone lactylation coordinate chromatin- and protein-level remodeling. This schematic summarizes representative functional outputs of histone and non-histone lactylation. **a**–**c** Non-histone lactylation regulates enzyme activity and metabolic flux, protein organization and signaling, and protein stability and trafficking. **d**–**f** Histone lactylation alters chromatin accessibility, promoter- and enhancer-associated transcription, and context-dependent transcriptional output. The illustrated mechanisms derive from distinct molecular, cellular, and disease settings and are not intended to occur simultaneously within a single cell; their effects depend on the modified substrate, lysine site, and local regulatory context. Orange dots represent lactate; orange “Kla” symbols and the suffix “la” denote lysine lactylation at the indicated residue; ↑ and ↓ indicate increases and decreases, respectively

The significance of protein lactylation lies in its ability to provide a molecular route through which local lactate pressure can be encoded. Elevated lactate may reflect metabolic remodeling within diseased lesions, but a lactylation-dependent mechanism is established only when lactate-associated changes generate modifications on specific proteins and lysine residues, and thereby alter target protein function, transcriptional programs, or cellular states [56, 57]. Current evidence indicates that lactylation can reshape transcriptional programs [58] and can also regulate immune function by modulating protein stability and signaling execution [57, 59]. According to the modified substrates, protein lactylation is generally classified into histone lactylation and non-histone lactylation [60]. Histone lactylation primarily affects chromatin states and transcriptional output [61, 62], whereas non-histone lactylation acts more directly on pre-existing functional proteins (Fig. 1d) [63, 64]. Representative chromatin- and protein-level outputs of histone and non-histone lactylation are summarized in Fig. 2.

### Biochemical formation and context-dependent control of lactylation

Protein lactylation is a post-translational modification in which lactate-derived lactyl groups are added to lysine residues on proteins [53]. This process involves lactate supply, lactyl-group activation, enzymatic transfer, and dynamic removal. Intracellularly generated lactate and extracellular lactate taken up by cells can contribute to the intracellular lactate pool available for lactylation. Lactate can then be activated through distinct enzymatic routes. Alanyl-tRNA synthetase 1/2 (AARS1/2) bind lactate, generate a lactyl-AMP intermediate, and directly catalyze lysine lactylation, whereas acetyl-CoA synthetase 2 (ACSS2) generates lactyl-CoA, which can serve as an activated lactyl donor for acyltransferase-dependent reactions [65, 66]. Writer enzymes subsequently transfer lactyl groups to lysine residues on proteins, generating lysine lactylation (Fig. 1c) [60].

Current attention has focused on writer mechanisms involving canonical lysine acyltransferases and aminoacyl-tRNA synthetase-related pathways. E1A-binding protein p300 (p300) was among the earliest proposed candidate writers of histone lactylation [9]. Its involvement suggests that lactylation shares part of its enzymatic machinery with other lysine acylations, such as acetylation, and that lactyl-CoA may compete with other acyl donors for shared enzymatic systems [67–69]. AARS1 can bind lactate and use ATP to generate a lactyl-AMP intermediate, followed by transfer of the lactyl group to lysine residues on proteins [70]. Further studies have identified AARS1 and AARS2 as intracellular L-lactate sensors and global lysine lactyltransferases, with AARS2 specifically mediating the lactylation and inactivation of cyclic GMP–AMP synthase (cGAS) [71]. These findings indicate that lactylation writing depends on route-specific lactyl-group activation, distinct enzymatic systems, and substrate accessibility.

Lactylation is also regulated by dynamic removal and modification competition. Several members of the histone deacetylase (HDAC) and sirtuin (SIRT) families have delactylase activity; for example, HDAC1–3 can function as histone lysine delactylases (Fig. 1c) [72]. Because these enzymes are not specific to lactylation alone, their enzymatic promiscuity creates potential crosstalk between lactylation and other lysine acylations through shared regulatory enzymes and, in some contexts, overlapping lysine substrates [68, 72]. Compared with writer and eraser systems, lactylation reader systems remain poorly defined. Only a small number of reader proteins have been experimentally identified, including DPF2 and the TRIM33 bromodomain [73, 74]. Although these studies have begun to define reader domains and functional outputs, the broader repertoire of lactylation readers, their site selectivity, and the context dependence of their downstream effects remain incompletely understood.

The formation of lactylation is highly context-dependent. Lactate concentration provides the substrate condition, but the specific lactylation landscape is also shaped by route-specific lactyl-group activation, writer and eraser activities, substrate accessibility, and crosstalk with other post-translational modifications (PTMs) [65, 68, 70–72]. The magnitude and duration of lactate exposure may influence the extent and persistence of lactylation; however, whether sustained exposure produces persistent transcriptional or protein-functional outputs depends on the modified substrate and cellular context [9, 53, 71]. Because individual lysine residues may be subject to alternative PTMs, including acetylation, methylation, ubiquitination, succinylation, and other modifications, the functional significance of lactylation should be interpreted within the broader PTM network [75–77].

### Detection strategies and evidence hierarchy for protein lactylation

Research on protein lactylation typically progresses from global signal detection to site identification and functional validation. Anti-Kla antibodies, immunoblotting, immunofluorescence, and immunoprecipitation can be used to assess overall lactylation levels or the modification status of candidate proteins. Precise site mapping generally relies on affinity enrichment of Kla-containing peptides followed by liquid chromatography–tandem mass spectrometry (LC–MS/MS), whereas subcellularly resolved lactylome profiling additionally requires prior subcellular fractionation [78, 79]. Mass spectrometry is particularly suited for screening lactylated substrates, identifying modification sites, and performing relative quantification, thereby advancing pan-Kla signals to the level of specific proteins and individual lysine residues (Fig. 1e) [78]. However, lactylation sites identified by mass spectrometry do not necessarily have functional significance, and the relationship between site occurrence and functional dependence requires further validation.

Determining whether a lactylation event is functionally meaningful requires establishing a causal link among site-specific modification, protein function, and cellular phenotype [56]. Lysine-site mutation can provide preliminary evidence that a given residue contributes to the observed functional alteration, but it does not by itself establish lactylation-specific causality [71]. Writer or eraser perturbation can test whether lactylation levels are regulated [65, 71, 72], whereas interaction assays, subcellular localization analyses, and functional assays of candidate proteins help determine whether the modification alters protein activity, stability, localization, molecular interactions, or downstream cellular outputs [56]. It should be noted that conventional K-to-Q mutations do not fully mimic authentic lactylation states [56, 80]. Moreover, perturbation of broadly acting writers or erasers may affect multiple lysine acylations or other cellular processes [68, 72]. These results should therefore be interpreted together with site-specific evidence.

Important limitations remain in current detection systems. Antibody-based assays may be affected by limited specificity and potential cross-reactivity with structurally related lysine modifications or lactylation isomers [53]. Bulk tissue or mixed-cell samples often cannot resolve cell-type-specific metabolic and lactylation-associated states, while conventional bulk measurements also lose lesion-level spatial and temporal context [81–83]. Spatial metabolic heterogeneity can further cause global measurements to obscure changes in critical cell populations or specific microenvironmental niches [84, 85]. Therefore, lactylation analysis should integrate cell-type resolution, spatial localization, and functional perturbation experiments, rather than directly inferring a pathogenic mechanism from an overall increase in lactylation.

On the basis of these limitations, this review stratifies evidence across the lactate–lactylation axis into three levels. The first level comprises metabolic stress readouts, including elevated lactate levels, increased acidification, changes in LDH or MCT expression, and enhanced pan-Kla signals; these indicators primarily define a lactate-stressed background. The second level comprises functional lactylation events, which require identification of the modified protein and lysine residue, together with evidence that the modification alters chromatin regulation, protein behavior, or downstream cellular phenotype. The third level comprises disease-dependent functional nodes, which require disease-relevant genetic or pharmacological evidence showing that selective disruption of the specific lactylation event attenuates or reverses the pathological phenotype (Fig. 1e).

## Histone lactylation in epigenetic gene regulation

Histone lactylation provides an important route through which lactate-associated metabolic states can be translated into chromatin regulation. Compared with elevated lactate levels or enhanced pan-Kla signals, the transcriptional consequences of histone lactylation depend more strongly on the modified histone residue, genomic location, local chromatin state, and surrounding epigenetic context. Histone lactylation should therefore be understood as a site- and locus-dependent mechanism of metabolic–epigenetic regulation.

### Histone lactylation marks and chromatin distribution

The initial study identified multiple lysine lactylation sites on core histones, including histone H3 lysine 18 lactylation (H3K18la), H3K23la, H3K27la, H3K9la, H4K5la, H4K8la, and H4K12la [9]. Distributed across the H3 and H4 tails, these marks constitute a heterogeneous chromatin modification landscape whose regulatory properties may vary according to the modified residue, genomic locus, and surrounding chromatin context. Subsequent characterization of these marks has progressed unevenly. H3K18la has been mapped most extensively at the genome-wide level, whereas other histone lactylation marks have mainly been investigated in more restricted cellular or pathological contexts [73, 86, 87]. H3K18la therefore currently provides one of the most developed frameworks for examining how lactate-linked histone modifications are distributed across chromatin.

Genome-wide profiling indicates that histone lactylation is not uniformly distributed across chromatin. H3K18la has been detected at active promoters, tissue-specific enhancers, and distal regulatory regions [87–89]. This distribution places H3K18la predominantly within transcriptionally permissive chromatin, but does not imply a uniform activating effect on transcription. Rather, its regulatory consequences depend on the occupied genomic element, the local chromatin state, and the cellular context [90, 91]. Accordingly, assessment of histone lactylation should focus on its genomic location, target-gene selectivity, and whether it is causally linked to changes in cellular state.

### Site- and locus-specific transcriptional consequences of histone lactylation

Histone lactylation influences transcription by shaping the local chromatin regulatory environment. As with other lysine acylations, lactylation neutralizes the positive charge of lysine and is therefore expected to weaken histone–DNA electrostatic interactions and favor a more permissive local chromatin configuration. Consistent with this model, lactylated regulatory regions show increased chromatin accessibility [90], whereas reducing lactate-dependent histone lactylation decreases accessibility at selected regulatory loci (Fig. 2d) [91, 92]. These observations provide a mechanistic basis for locus-restricted transcriptional regulation, although direct causality should be established through site-specific and locus-specific perturbation.

Transcriptional regulation mediated by histone lactylation is temporally controlled and gene-selective. In models of M1 macrophage polarization, lactylation increases during the late phase of polarization and is associated with the expression of homeostatic or repair-related genes such as Arg1 [9]. In models of myocardial infarction and ischemia-induced muscle regeneration, H3K18la in monocytes or macrophages increases within defined injury windows, its genomic enrichment changes along disease progression, and its distribution can predict subsequent changes in gene expression [88, 93]. In a bacillus Calmette–Guérin (BCG)-induced trained immunity model, H3K18la persists after removal of the primary stimulus and is associated with enhanced inflammatory cytokine responses upon restimulation [89]. These findings suggest that histone lactylation can convert fluctuations in lactate metabolism into chromatin states with gene selectivity and temporal memory (Fig. 2e).

Studies of other histone lactylation marks further indicate that the modified residue alone does not determine a fixed transcriptional outcome. The same mark can regulate distinct target genes and biological effects depending on the cellular and pathological context. For example, reduction of BRD4-dependent H4K8la aggravated maladaptive A1 astrocyte polarization after subarachnoid hemorrhage [94], whereas H4K8la promoted fibroblast activation protein (FAP) transcription in gastric cancer mesenchymal stromal cells [95] and bone morphogenetic protein 7 (BMP7) transcription in esophageal squamous cell carcinoma [96]. Histone lactylation can also reinforce lactate production through positive-feedback regulation, as H4K5la-mediated enolase 2 (ENO2) transcription further enhances glycolysis and lactate production [97]. These findings indicate that the transcriptional consequences of histone lactylation depend on the occupied genomic locus, local chromatin environment, and regulatory machinery available in the responding cell, rather than on the modified residue alone (Fig. 2f). However, systematic comparisons of the genome-wide distribution, temporal dynamics, and functional dependence of these marks remain limited. Selected examples illustrating the context-dependent consequences of these histone lactylation marks are provided in Table 1.

**Table 1:** Representative histone lactylation events in inflammatory and tumor microenvironments

Mark	Biological context (cell type)	Main functional consequence	Reference
H2BC9 K44la	Esophageal squamous cell carcinoma (cancer cells)	Enhances WNT7B transcription and activates Wnt/β-catenin signaling, thereby promoting esophageal squamous cell carcinoma proliferation and tumor growth	[98]
H3K9la	Rheumatoid arthritis (fibroblast-like synoviocytes)	Promotes NFATC2 transcription and supports inflammatory activation of fibroblast-like synoviocytes	[99]
H3K14la	Sepsis-induced acute lung injury (pulmonary endothelial cells)	Promotes endothelial ferroptosis and vascular dysfunction via TFRC/SLC40A1	[100]
	Cervical cancer (cancer cells)	Enables DPF2-driven oncogenic transcription and cell survival	[73]
H3K18la	LPS/IFN-γ-induced M1 polarization (M1 macrophage)	Associated with late-phase transcriptional activation of Arg1 and other repair/homeostatic genes	[9]
	Myocardial infarction (monocytes/macrophages)	Activates Lrg1, Vegf-a and Il-10, supporting repair and angiogenesis	[93]
	Small-cell lung cancer (naïve CD8⁺ T cells)	Activates Nur77 and enhances tonic T-cell receptor signaling, reducing antigen responsiveness	[101]
H3K23la	Hypertrophic scar (dermal fibroblasts)	Activates HEY2 and COL11A1 transcription and establishes an MCT1-dependent profibrotic feedback loop	[102]
H3K27la	Gastric adenocarcinoma (cancer cells)	Enhances PSMD14 and SOX9 transcription, reinforcing glycolysis and tumor stemness	[103]
H4K5la	Hepatocellular carcinoma (cancer cells)	Promotes ENO2 transcription and reinforces glycolysis–lactate production	[97]
H4K8la	Subarachnoid hemorrhage (astrocytes)	Restrains maladaptive A1 astrocyte polarization after subarachnoid hemorrhage	[94]
	Gastric cancer (GCMSCs)	Promotes FAP transcription, ECM remodeling and CD8⁺ T-cell exclusion	[95]
	Esophageal squamous cell carcinoma (cancer cells)	Promotes BMP7 transcription and supports esophageal squamous cell carcinoma progression	[96]
H4K12la	Psoriasis (keratinocytes)	Promotes IL-17A-driven keratinocyte proliferation through the KLK8–IL-17R–HAT1 loop	[104]
	Colorectal cancer (CCSCs)	Upregulates GCLC, suppresses ferroptosis and promotes chemoresistance	[105]
	Ovarian cancer (cancer cells)	Promotes PGK1 transcription and glycolytic flux, thereby driving ovarian cancer progression	[106]
H4K79la and H4K91la	Breast cancer (cancer cells)	Promote glycolytic-gene transcription and reinforce a glycolysis–lactylation positive-feedback loop, thereby supporting breast cancer progression	[107]

### Epigenetic crosstalk shaping histone lactylation outputs

Histone lactylation should be interpreted within the broader network of chromatin modifications. Histone lysine residues can undergo canonical modifications such as acetylation, methylation, and ubiquitination, as well as metabolite-sensitive acylations including lactylation, crotonylation, and succinylation [108, 109]. At the same lysine residue, these modifications generally represent alternative and potentially competing states, whereas modifications at neighboring residues can form combinatorial patterns that influence histone-tail charge, protein-interaction interfaces, reader recruitment, and local chromatin conformation [110–112]. Therefore, an increase in a single lactylation site does not directly predict a fixed transcriptional outcome. Its functional direction must be evaluated in relation to the chromatin region in which it occurs, the combination of neighboring modifications, and the expression changes of target genes.

Lactylation shares particularly close mechanistic parallels with acetylation. Both are lysine acylation modifications and may exhibit crosstalk through shared regulatory enzymes, context-dependent acyl-donor availability, and overlapping lysine substrates [68, 113]. Both modifications can be detected in active chromatin regions, but current evidence suggests that lactylation and acetylation differ in the timing of transcriptional responses and in target-gene preference. They are therefore better viewed as complementary components of a metabolically sensitive acylation network [9, 114]. Accordingly, analyses of H3K18la or other Kla marks should assess their relationship with co-occurring acetylation marks and determine whether these combinations contribute to target-gene selectivity.

The relationship between histone lactylation and methylation is currently supported mainly by colocalization analyses and a limited number of mechanistic studies. H3K18la often positively correlates with H3K4me3, H3K27ac, and gene expression, suggesting that it is frequently present in transcriptionally permissive chromatin environments [87]. However, in retinoblastoma, lactylation has also been linked to the polycomb repressive complex 2 (PRC2)–SUZ12 axis, enrichment of H3K27me3 at the KLF4 promoter, and transcriptional repression of KLF4 [115]. Together, these findings indicate that the functional direction of histone lactylation depends on the specific chromatin context. Interpretation should therefore move beyond whether H3K18la or pan-Kla signals are increased, and instead focus on genomic loci, target-gene selectivity, neighboring epigenetic modifications, and transcriptional consequences.

## Non-histone lactylation in the regulation of protein function

Non-histone lactylation extends lactylation research beyond chromatin regulation to the direct control of protein function. Unlike histone lactylation, which primarily affects transcriptional programs, non-histone lactylation occurs directly on pre-existing functional proteins and can therefore exert relatively direct effects on protein behavior and downstream cellular processes. This class of modification can alter protein activity, stability, subcellular localization, and molecular interactions [60]. Through these effects, non-histone lactylation can link local lactate pressure to metabolic adaptation, immune regulation, stress responses, and therapy resistance (Fig. 2a–c). Accordingly, interpretation of non-histone lactylation should not focus merely on the number of substrates or the scale of identified Kla sites, but should clarify which sites have verifiable consequences for protein function and pathological relevance.

### Substrate diversity and site-specific interpretation of non-histone lactylation

Current evidence indicates that non-histone lactylation substrates are broadly distributed across functional modules, including metabolic enzymes, DNA damage repair proteins, tumor suppressors, and innate immune molecules [57]. Compared with the relatively limited set of modification sites identified on core histones, non-histone lactylation sites are substantially more numerous and broadly distributed across cellular proteins [116, 117]. Their functional consequences depend on the specific substrate, modified residue, and structural context, including whether the modification alters catalytic activity, molecular interactions, complex assembly, subcellular localization, or protein stability [56, 57, 70, 71, 118, 119].

Existing studies provide direct evidence for this substrate diversity. Lactylome profiling of colorectal cancer SW480 cells and hepatocellular carcinoma tissues has shown that non-histone lactylation can extensively intersect with core carbon metabolism, amino acid metabolism, lipid metabolism, and nucleotide metabolism networks [116, 117]. Site-focused studies of substrates such as NBS1, MRE11, p53, and cGAS further indicate that non-histone lactylation has expanded beyond metabolic enzymes into functional networks governing DNA repair, tumor suppression, and innate immunity [70, 71, 118, 119]. These findings suggest that the key question in non-histone lactylation research has shifted from whether this modification exists to which sites produce functional consequences.

### Functional consequences of non-histone lactylation

Non-histone lactylation can produce distinct functional consequences depending on the structural context of the modified site. Regulation of enzymatic activity is one of the more clearly defined mechanisms. In colorectal cancer SW480 cells, lactylation of platelet-type phosphofructokinase (PFKP) at K688 weakens its enzymatic activity [117]. In the hepatocellular carcinoma lactylome, lactylation of adenylate kinase 2 (AK2) at K28 inhibits AK2 enzymatic activity and is associated with hepatocellular carcinoma cell proliferation and metastasis [116]. These findings indicate that site-specific lactylation can directly alter metabolic enzyme function, thereby affecting metabolic flux and tumor cell behavior (Fig. 2a).

Non-histone lactylation can also regulate protein stability and degradation routes. Programmed death-ligand 1 (PD-L1) K271 lactylation shifts PD-L1 trafficking toward Rab11-positive recycling endosomes and away from Rab7b- and LAMP1-positive degradative compartments, thereby reducing lysosomal degradation and maintaining PD-L1 stability and cell-surface abundance [120]. This finding extends immune checkpoint research from transcriptional regulation to protein stability and membrane trafficking. Similarly, lactylation of hypoxia-inducible factor 1α (HIF-1α) at species-specific residues—K644 in mice and K12 in humans and pigs—impairs von Hippel–Lindau (VHL) recognition, reduces K48-linked ubiquitination and proteasomal degradation, and thereby enhances HIF-1α stability [121]. These findings suggest that lactylation may increase the availability of key functional proteins by modulating protein recycling and degradation processes (Fig. 2c).

Non-histone lactylation can also alter protein spatial organization and signaling output. In hepatitis B virus (HBV)-associated hepatocellular carcinoma, lactylation of spectrin alpha, non-erythrocytic 1 (SPTAN1) at K1952 and K1957 disrupts its cytoplasmic liquid–liquid phase separation and promotes its nuclear translocation. Once in the nucleus, lactylated SPTAN1 interacts with core-binding factor subunit beta (CBFB) and activates notch receptor 1 (NOTCH1)/HES family bHLH transcription factor 1 (HES1) signaling [122]. In innate immune signaling, AARS2-mediated lactylation of human cGAS at K131 weakens cGAS liquid–liquid phase separation, DNA binding, and enzymatic activity, thereby suppressing cGAS–stimulator of interferon genes (STING) signaling [71]. Together, these findings indicate that lactylation can regulate cellular responses that depend on signaling-platform assembly by altering protein localization, condensate formation, and molecular interactions (Fig. 2b). Such regulation can alter signaling output without necessarily requiring changes in total protein abundance, providing a route through which elevated lactate influences stress responses, innate immune recognition, and tumor adaptation. Representative non-histone lactylation events, including their substrates, modification sites, and functional outputs, are summarized in Table 2.

**Table 2:** Representative non-histone lactylation events in inflammatory and tumor microenvironments

Mark	Biological context (cell type)	Main functional consequence	Reference
cGAS K131la (human)/K156la (mouse)	High-lactate viral infection and stress models (PBMCs, BMDMs and peritoneal macrophages)	Impairs cGAS phase separation, DNA binding and cGAMP synthesis, thereby suppressing cGAS–STING signaling	[71]
HMGB1 lactylation	Sepsis-associated kidney injury (macrophages)	Promotes NET formation via cGAS–STING signaling	[123]
NLRP3 K24la/K565la	Inflammasome activation (macrophages/inflammatory cells)	Facilitates ASC recruitment and inflammasome assembly	[124]
S100A9 K26la	Myocardial ischemia/reperfusion injury (neutrophils)	Promotes neutrophil trafficking and cardiac inflammation	[125]
PKM2 K62la	Pro-inflammatory macrophage model (macrophages)	Reprograms macrophage metabolism toward a repair-associated phenotype	[126]
PFKP K688la	Colorectal cancer (SW480 cells)	Reduces PFKP enzymatic activity	[117]
AK2 K28la	Hepatocellular carcinoma (cancer cells)	Inhibits AK2 kinase activity and supports hepatocellular carcinoma cell proliferation and metastasis	[116]
HNRNPA1 K350la	Bladder cancer (cancer cells)	Promotes PKM pre-mRNA splicing toward PKM2 and reinforces glycolytic reprogramming	[127]
TFEB K91la	Pancreatic cancer (cancer cells)	Blocks WWP2-mediated ubiquitination, stabilizes TFEB and enhances autophagy–lysosomal activity	[128]
eEF1A2 K408la	Colorectal cancer (cancer cells)	Enhances translation elongation and supports tumor growth in a high-lactate microenvironment	[63]
HIF-1α K172la	Esophageal squamous cell carcinoma (cancer cells)	Promotes HIF-1α/HIF-1β assembly and hypoxia-mediated immune evasion	[129]
SPTAN1 K1952la/K1957la	HBV-related hepatocellular carcinoma (cancer cells)	Promotes SPTAN1 nuclear translocation and NOTCH1/HES1 activation	[122]
ENSA K63la	Pancreatic ductal adenocarcinoma (cancer cells)	Activates SRC/STAT3/CCL2 signaling and macrophage-mediated immune suppression	[130]
MRE11 K673la	DNA-damaging therapy (cancer cells)	Enhances DNA binding and HR repair	[119]
NBS1 K388la	Cisplatin-resistant gastric cancer (cancer cells)	Promotes MRN complex assembly and homologous recombination repair, thereby conferring chemotherapy resistance	[118]
KU70 K317la	Glioblastoma GSC-TAM niche (GSCs)	Suppresses cGAS–STING signaling and promotes immune escape	[131]
PD-L1 K280la	Non-small-cell lung cancer (cancer cells)	Stabilizes PD-L1 by blocking HUWE1-mediated ubiquitination	[132]
PD-L1 K271la	Colorectal cancer (cancer cells)	Lactylation promotes tumor immune evasion by stabilizing PD-L1 protein	[120]
CD44 K158la (human)/K163la (mouse)	KRAS-mutant colorectal cancer (CD8⁺ T cells)	Weakens hyaluronan binding and AKT signaling, limiting CD8⁺ T-cell antitumor function	[133]
APOC2 K70la	Non-small-cell lung cancer (cancer cells)	Stabilizes APOC2, promoting free fatty acid release, Treg accumulation and immune checkpoint inhibitor resistance	[134]

### Integration of histone and non-histone lactylation with other post-translational modifications

Histone lactylation and non-histone lactylation represent two complementary layers of lactate signaling output [135]. Histone lactylation primarily shapes cellular states through chromatin-associated transcriptional regulation and, in some contexts, can support persistent epigenetic programs [89]. Non-histone lactylation modifies pre-existing functional proteins and can alter their activity, stability, localization, or molecular interactions without requiring de novo transcription [136]. These two modes of lactylation can coexist within the same cell and jointly determine the final phenotype after lactate exposure, although current evidence is not sufficient to support a fixed temporal sequence between them.

Non-histone lactylation should also be interpreted within the broader post-translational modification network. Non-histone proteins are extensively regulated by lysine acetylation, ubiquitination, methylation, SUMOylation, and other PTMs [137]. Studies of HIF-1α lactylation and transcription factor EB (TFEB) K91 lactylation show that lactylation can enhance target protein stability by blocking recognition by E3 ubiquitin ligases or weakening ubiquitin-mediated degradation [121, 128]. Therefore, the function of lactylation depends not only on the modification itself, but also on how it alters the recognition of the substrate by other PTM-regulatory enzymes and thereby reshapes downstream modification states.

Overall, the central value of non-histone lactylation lies in its ability to direct lactate signals onto functional proteins. Through site-dependent mechanisms, non-histone lactylation can regulate metabolic enzyme activity, protein stability, degradation routes, spatial distribution, and signaling-complex assembly. Its functional direction depends on the target protein, the position of the modified residue, and the pathological context. Its pathological significance should therefore be assessed through site-level validation and functional perturbation experiments.

## Lactate and lactylation in inflammatory microenvironments

Lactate metabolism and lactylation in inflammatory microenvironments are characterized by pronounced stage dependence, plasticity, and cell-context specificity. Infection or tissue injury can induce immune-cell infiltration, restrict local oxygen delivery, and activate structural cells, collectively enhancing glycolytic flux and increasing local lactate burden. Lactate availability changes across inflammatory initiation, injury amplification, repair, and resolution, and may generate distinct metabolic, signaling, and lactylation-dependent outputs at different stages. Accordingly, the lactate–lactylation axis in inflammation should not be classified simply as pro-inflammatory or anti-inflammatory. Its biological significance should instead be interpreted according to the inflammatory stage, responding cell type, spatial niche, mode of lactate action, and, where lactylation is implicated, the modified substrate and site.

### Dynamic lactate exposure across inflammatory niches

**Fig. 3:**
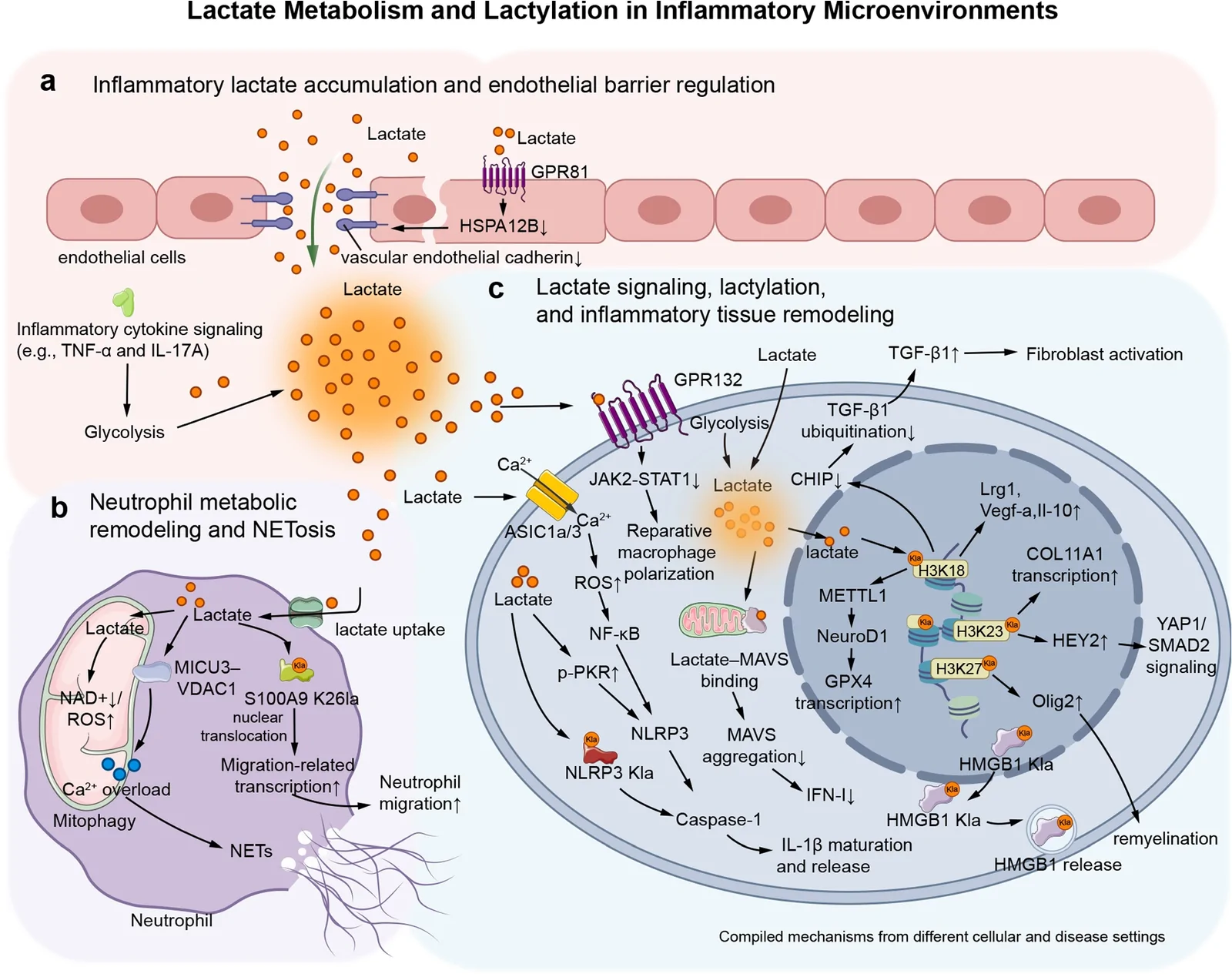
Regulatory roles of lactate metabolism and lactylation in inflammatory microenvironments. This schematic summarizes representative lactate- and lactylation-dependent mechanisms across inflammatory stages and tissue contexts. **a** Cytokine-driven glycolysis promotes local lactate accumulation, whereas endothelial GPR81 signaling regulates barrier integrity. **b** In neutrophils, lactate uptake disrupts mitochondrial Ca^2^⁺ and redox homeostasis, promoting mitophagy and NETosis, whereas S100A9 K26la enhances migration-related transcription. **c** Across distinct inflammatory cell and tissue contexts, lactate-responsive receptors, direct MAVS binding, and histone or non-histone lactylation regulate macrophage polarization, inflammasome and type I interferon signaling, fibroblast activation, tissue remodeling, remyelination, and HMGB1 release. These mechanisms derive from different disease settings and are not intended to occur simultaneously within a single cell. The prefix “p-” denotes phosphorylation. Orange dots represent lactate; orange “Kla” symbols and the suffix “la” denote lysine lactylation at the indicated residue; ↑ and ↓ indicate increases and decreases, respectively

Elevated lactate in inflammatory microenvironments is not driven solely by hypoxia, but is also promoted by cellular metabolic reprogramming and amplification of inflammatory cytokine signaling [138, 139]. During inflammatory activation, macrophages, neutrophils, and T cells can adopt glycolysis-dependent metabolic programs and contribute to local lactate production [25, 140–142]. In chronic inflammatory tissues, cytokine-driven metabolic reprogramming can sustain a glycolytic, lactate-producing microenvironment. TNF-α promotes GLUT1/HIF-1α-dependent glycolysis in rheumatoid synovial fibroblasts [143], whereas IL-17A enhances HIF-1α-dependent epithelial glycolysis and lactate production in psoriatic skin (Fig. 3a) [144].

Lactate burden is often associated with inflammatory activity and tissue status. In rheumatoid arthritis, elevated synovial lactate concentrations and lactate-associated metabolic signals are linked to greater local inflammatory and pathological severity [145, 146], and in ulcerative colitis, fecal lactate concentrations increase with disease activity and severity [47]. Changes in lactylation can also reflect inflammatory status. In critically ill patients, peripheral blood mononuclear cell H3K18la levels are highest in those with septic shock and positively correlate with Acute Physiology and Chronic Health Evaluation II (APACHE II) scores, day-1 Sequential Organ Failure Assessment (SOFA) scores, and serum lactate levels [147]. In rheumatoid arthritis, global lactylation is increased in fibroblast-like synoviocytes, and autoantibodies against lactylated histones positively correlate with Disease Activity Score in 28 joints (DAS28) [99].

The lactate–lactylation axis in inflamed tissues shows clear spatial and cell-type specificity. Single-cell transcriptomic analysis of Crohn’s disease showed higher lactylation-related gene-set scores in immune cells from inflamed sites than in those from non-inflamed regions [148]. In fibrotic lung lesions, lactate derived from myofibroblasts can induce histone lactylation in macrophages within local fibrotic niches [149]. At the modification-site level, H3K9la is markedly increased in rheumatoid arthritis fibroblast-like synoviocytes (RA-FLS) [99], and the distribution of H3K18la and H3K9la across distinct CD8⁺ T-cell states is also associated with metabolic features and functional status [62]. These findings indicate that the inflammatory lactylation landscape is jointly shaped by pathological region, cell type, and modification site.

### Lactate-mediated metabolic and inflammatory remodeling

Lactate can reshape inflammatory responses through metabolic reutilization, but its functional effects vary with concentration and cellular context. A study of trained immunity showed that blocking lactate uptake or metabolism weakens the capacity of β-glucan-trained monocytes to produce TNF-α and IL-6, suggesting that lactate can serve as a tricarboxylic acid (TCA)-cycle carbon source for subsets of inflammatory cells [92]. Under pH-neutral conditions, lactate can be taken up by activated CD8⁺ T cells and used as a carbon source [150, 151]. Conversely, excessive lactate can restrict T-cell proliferation independently of extracellular acidification by shifting the NAD⁺/NADH balance toward NADH and limiting NAD⁺-dependent metabolic reactions [152]. Lactic acidosis imposes a distinct constraint on cytotoxic T lymphocytes by blocking cytokine production, including interferon-γ (IFN-γ), and partially impairing lytic granule exocytosis [153]. Lactate can also support regulatory or reparative programs in selected immune-cell states. It enhances oxidative phosphorylation and suppressive function in regulatory T cells (Tregs) [154], whereas low-dose lactate promotes an oxidative phosphorylation-dependent shift toward reparative macrophage states and facilitates peripheral nerve repair; excessive lactate instead induces mitochondrial stress without regenerative benefit [155].

Lactate can also modulate inflammatory progression through receptor signaling and intracellular metabolic perturbation. In sepsis-associated endothelial injury, lactate promotes vascular hyperpermeability through GPR81-dependent suppression of heat shock protein family A member 12B (HSPA12B) and disruption of endothelial junctional integrity (Fig. 3a) [156]. During the recovery phase of acute pancreatitis, lactate suppresses Janus kinase 2 (JAK2)–signal transducer and activator of transcription 1 (STAT1) signaling through G protein-coupled receptor 132 (GPR132), reduces pro-inflammatory gene expression, and promotes macrophage transition toward a reparative phenotype [157]. Lactic acid fermentation can promote NOD-, LRR- and pyrin domain-containing protein 3 (NLRP3) inflammasome activation through lactate-dependent protein kinase R (PKR) phosphorylation [158]. In nucleus pulposus cells, extracellular lactate activates acid-sensing ion channels 1a and 3 (ASIC1a and ASIC3)-dependent Ca^2^⁺ influx and downstream ROS–NF-κB signaling, thereby promoting NLRP3 activation, caspase-1 activity, IL-1β release, and pyroptosis [159]. Lactate can directly bind the transmembrane domain of mitochondrial antiviral-signaling protein (MAVS) and prevent its aggregation, thereby suppressing signaling by retinoic acid-inducible gene I-like receptors (RLRs) and downstream type I interferon (IFN-I) production (Fig. 3c) [160].

At the tissue-effector level, lactate can promote neutrophil extracellular trap (NET) formation through mitochondrial metabolic and calcium-dependent mechanisms, with context-dependent consequences for host defense and tissue injury. In a myocardial ischemia–reperfusion model, lactate increases mitochondrial calcium uptake 3 (MICU3) abundance in neutrophils and promotes the interaction of MICU3 with voltage-dependent anion channel 1 (VDAC1), leading to excessive mitochondrial Ca^2^⁺ uptake, mitochondrial dysfunction, mitophagy activation, and NET formation [161]. In infectious inflammation, *Staphylococcus aureus*-derived lactate is transported from phagosomes to neutrophil mitochondria, where its oxidation to pyruvate depletes mitochondrial NAD⁺ and increases mitochondrial reactive oxygen species, thereby triggering NETosis (Fig. 3b) [162]. Thus, lactate is not merely a consequence of inflammatory metabolic reprogramming but can also act as a metabolic signal that drives context-dependent neutrophil effector responses and, under sterile inflammatory conditions, aggravates tissue injury.

### Lactylation in inflammatory persistence and tissue repair

Beyond its roles in metabolic utilization, extracellular acidification, and receptor signaling, lactate can regulate inflammatory states through lactylation-dependent transcriptional and protein-functional changes. Histone lactylation mainly influences the expression of inflammation-related genes, whereas non-histone lactylation more directly regulates the functions of inflammatory mediators, structural proteins, metabolic enzymes, and signaling proteins. Lactylation in inflammation should therefore not be rigidly defined as either pro-inflammatory or anti-inflammatory; its functional direction depends on disease stage, cell type, and the molecular target and modified site [139].

Histone lactylation can translate lactate pressure during inflammation into cell-state transitions and tissue-remodeling programs (Fig. 3c). In arsenite-induced liver fibrosis, hepatocyte-derived lactate increases H3K18la-dependent interferon regulatory factor 4 (IRF4) transcription in CD4⁺ T cells, thereby promoting T helper 17 (Th17) cell differentiation and IL-17A-mediated hepatic stellate cell activation [163]. In rheumatoid arthritis, elevated lactate in the synovial microenvironment increases p300-dependent H3K18la in rheumatoid arthritis synovial fibroblasts. H3K18la promotes methyltransferase 1 (METTL1) expression, after which METTL1 stabilizes neuronal differentiation 1 (NeuroD1) mRNA through m^7^G modification and NeuroD1 enhances glutathione peroxidase 4 (GPX4) transcription, thereby promoting ferroptosis resistance and synovial fibroblast hyperplasia [164].

Histone lactylation can also support tissue repair. In myocardial infarction models, general control nonderepressible 5 (GCN5)-mediated H3K18la in monocytes and macrophages activates repair-related genes such as Lrg1, Vegf-a, and Il-10, thereby promoting anti-inflammatory responses and angiogenesis [93]. After spinal cord injury, lactate-induced H3K27la increases expression of oligodendrocyte transcription factor 2 (Olig2) in oligodendrocyte precursor cells and promotes their differentiation, remyelination, and motor recovery [165].

Conversely, lactate-dependent histone lactylation can drive pathological tissue remodeling. In PM2.5-induced pulmonary fibrosis, increased lactate production in macrophages promotes H3K18la enrichment at the Stub1 promoter and suppresses expression of C terminus of Hsc70-interacting protein (CHIP), thereby reducing transforming growth factor-β1 (TGF-β1) ubiquitination and degradation, increasing its secretion, and exacerbating pulmonary fibrosis [166]. In hypertrophic scars, macrophage-derived lactate is taken up by fibroblasts through MCT1, which increases H3K23la at the promoters of the profibrotic genes HEY2 and COL11A1, thereby promoting profibrotic transcription and scar formation [102]. Together, these findings indicate that the function of histone lactylation is determined by the target gene program and the stage of disease.

Non-histone lactylation provides a more direct link between lactate exposure and the function of inflammatory regulatory and effector proteins. Some events can amplify inflammatory activation and tissue injury. For example, lactate induces HMGB1 lactylation and exosomal HMGB1 release from macrophages, thereby promoting downstream NET formation [123]. AARS2-mediated NLRP3 lactylation facilitates ASC recruitment and inflammasome activation [124], and S100 calcium-binding protein A9 (S100A9) K26 lactylation promotes neutrophil migration and cardiac recruitment and, following its release during NET formation, contributes to cardiomyocyte injury (Fig. 3b,c) [125]. Other lactylation events participate in pathogen clearance, negative-feedback regulation, and reparative transitions. Rubicon autophagy regulator (RUBCN) K33 lactylation enhances microtubule-associated protein 1 light chain 3 (LC3)-associated phagocytosis [167], nonenzymatic D-lactylation of RelA at K310 attenuates NF-κB transcriptional activity [168], and pyruvate kinase M2 (PKM2) K62 lactylation promotes macrophage reparative polarization [126]. Together, these findings indicate that non-histone lactylation exerts bidirectional and context-dependent effects in inflammation. Its pathological significance should therefore be assessed by distinguishing correlative changes in protein lactylation from site-specific modifications with demonstrated causal functions.

## Lactate and lactylation in tumor microenvironments

Elevated lactate in tumors is not a simple readout of hypoxia, but reflects the combined effects of oncogenic signaling-driven metabolic adaptation and multicellular lactate production. Tumor cells can sustain high glycolytic flux under both hypoxic and non-hypoxic conditions, while aberrant vasculature and uneven perfusion accentuate spatial gradients of hypoxia and lactate, leaving local tissues exposed to persistent lactate enrichment and acidic stress [45, 169]. In this context, lactate is not only a consequence of metabolic pressure, but also participates in shaping tumor ecology through metabolic exchange, stromal remodeling, immunosuppression, and lactylation-dependent modifications [170, 171].

### Lactate-rich niches and lactylation-prone tumor states

**Fig. 4:**
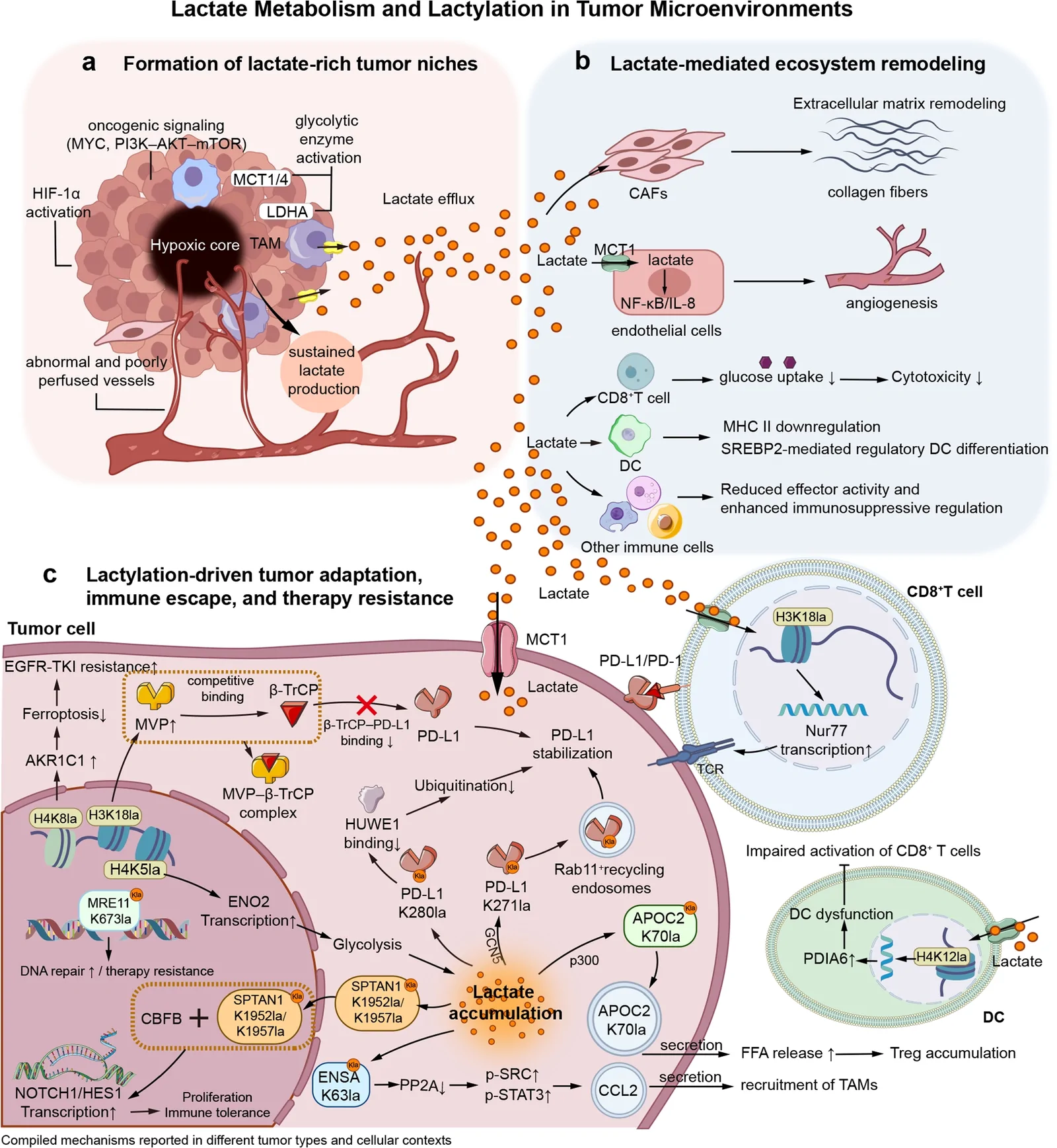
Regulatory roles of lactate metabolism and lactylation in the tumor microenvironment. This schematic summarizes the formation of lactate-rich tumor niches and their structural, immune, and lactylation-dependent consequences. **a** Hypoxia, oncogenic signaling, enhanced glycolysis, abnormal perfusion, and lactate transport jointly sustain intratumoral lactate accumulation and efflux. **b** Extracellular lactate acts on CAFs and endothelial cells to promote extracellular matrix remodeling and angiogenesis, while impairing CD8⁺ T-cell and DC function and reinforcing immunosuppressive regulation in other immune cells. **c** Across distinct tumor types and cellular contexts, histone and non-histone lactylation regulate DNA repair, glycolysis, proliferative signaling, immune checkpoint stability, immune-cell dysfunction, and immunosuppressive-cell recruitment. The illustrated mechanisms are not intended to occur simultaneously or form a single continuous pathway. The prefix “p-” denotes phosphorylation. Orange dots represent lactate; orange “Kla” symbols and the suffix “la” denote lysine lactylation at the indicated residue; ↑ and ↓ indicate increases and decreases, respectively

Tumor lactate enrichment is sustained by cancer-cell metabolic programs and metabolic contributions from stromal and immune compartments (Fig. 4a). Intratumoral hypoxia stabilizes HIF-1α and induces glucose transporters, glycolytic enzymes, and LDHA, thereby increasing glycolytic flux and lactate production [172], while oncogenic signaling involving MYC, phosphoinositide 3-kinase (PI3K), AKT, and mechanistic target of rapamycin (mTOR) can also enhance glycolytic flux [173, 174]. Microenvironmental cells, including tumor-associated macrophages (TAMs) and cancer-associated fibroblasts (CAFs), can further contribute to local lactate production and accumulation [131, 175, 176]. Thus, elevated lactate in tumors is not a single readout of hypoxia, but the integrated result of hypoxic signaling, oncogenic pathways, metabolic adaptation, and multicellular lactate sources [177].

Tumor lactate exposure is spatially heterogeneous. Uneven vascular perfusion, oxygen gradients, and differences in cellular composition can generate heterogeneous lactate distribution across tumor regions [45, 178]. Integrated spatial multi-omics in lung adenocarcinoma revealed cell-type-restricted lactate distributions and linked high-lactate tissues to stromal, vascular, and immune reprogramming [4], and dynamic metabolic imaging of glioblastoma has revealed higher lactate concentrations and glycolytic flux in tumor regions than in adjacent non-tumor or peritumoral brain regions [179]. Multi-omics analysis of lung adenocarcinoma further showed that epithelial cells and fibroblasts are increased in high-lactate tumors, whereas T/natural killer (NK) cells and monocyte–macrophage populations are more enriched in low-lactate tumors [4]. These findings suggest that spatial lactate gradients can help organize tumor niches along a metabolic–stromal–vascular–immune axis.

Lactate metabolism is also closely associated with tumor progression and invasive disease states. Elevated intratumoral lactate concentrations can predict subsequent lymph-node or distant metastasis in patients with head and neck cancer, and clinical studies across several primary carcinomas have linked lactate levels to metastatic potential and survival outcomes [180, 181]. Persistent intratumoral lactate accumulation can support tumor lactylation, which may itself exhibit spatial heterogeneity. For example, SPTAN1 lactylation is higher in HBV-positive hepatocellular carcinoma than in adjacent non-tumor tissues, whereas in glioblastoma, malignant cells with high transcriptome-derived lactylation-signature scores are preferentially enriched in hypoxic tumor cores relative to peri-tumoral regions [84, 122]. Clinically oriented studies also suggest that elevated H3K18la or pan-Kla signals are associated with tumor progression in certain cancers [182, 183].

This spatial heterogeneity indicates that lactate does not drive downstream responses uniformly across tumor tissues. High-lactate microenvironments are more likely to coactivate hypoxia-, angiogenesis-, and stromal remodeling-related signaling while providing a sustained substrate background for histone and non-histone lactylation. Low-lactate regions may retain greater immune-cell representation and potentially less suppressed immune states. Accordingly, tumor lactate gradients can generate distinct signaling-pathway activation patterns and lactylation outputs across different ecological niches, highlighting pronounced spatial and temporal complexity. These findings suggest that lactylation-associated transcriptional states may exhibit regional heterogeneity in GBM, although direct spatial validation of protein- and site-specific lactylation remains necessary.

### Lactate-driven remodeling of tumor ecosystems

Sustained lactate enrichment can reshape tumor ecology through metabolic exchange, structural remodeling, and immune regulation (Fig. 4a,b). Hypoxic or highly glycolytic cells typically export lactate through MCT4, whereas tumor cells with oxidative metabolic capacity can import lactate through MCT1 and incorporate it into mitochondrial metabolism, thereby establishing lactate shuttling in tumor tissues with uneven oxygen supply [5, 184]. In pancreatic ductal adenocarcinoma, CAFs can also take up tumor-derived lactate through MCT1 and use it in the tricarboxylic acid cycle to sustain their own functions, further supporting a fibrotic and immunosuppressive microenvironment [185]. Together, these forms of intercellular lactate transfer and reutilization can help sustain metabolic heterogeneity and adaptation to spatially heterogeneous oxygen and nutrient availability.

At the structural level, lactate enrichment and the associated acidic pressure can promote extracellular matrix (ECM) degradation, angiogenesis, and invasive stromal remodeling (Fig. 4b). An acidic microenvironment can enhance protease activity and ECM degradation, thereby facilitating basement-membrane breakdown and local invasion [186]. Lactate can also act directly on vascular endothelial cells. After endothelial cells take up lactate through MCT1, lactate can activate NF-κB/IL-8 and HIF-1-related pro-angiogenic signaling; blockade of MCT1 suppresses lactate-induced HIF-1 activation and tumor angiogenesis [33, 187]. In addition, CAF-derived lactate can promote the expression of collagen-family genes in prostate cancer cells, enhance cancer cell-autonomous ECM production and collagen remodeling, and support the formation of a pro-invasive microenvironment [188].

At the immune level, tumor-derived lactate generally weakens antitumor immunity and can simultaneously affect effector killing, antigen presentation, and myeloid regulation (Fig. 4b). Lactate accumulation accompanied by extracellular acidification can restrict glucose uptake by CD8⁺ T cells, reduce their proliferation and cytotoxic function, suppress the production of effector molecules such as IFN-γ and granzymes, and impair the antitumor activity of NK cells [189–191]. In a prostate cancer model, lactate shifts CD4⁺ T-cell responses from antitumor Th1 states toward regulatory T-cell states [192]. Lactate can also reduce major histocompatibility complex class II (MHC II) expression through a GPR81-dependent pathway involving cyclic adenosine monophosphate (cAMP) and class II major histocompatibility complex transactivator (CIITA), thereby impairing the antigen-presenting capacity of dendritic cells [193], and can drive conventional dendritic cells (DCs) toward mature regulatory DCs through sterol regulatory element-binding protein 2 (SREBP2) activation [194]. In endometrial cancer, tumor-derived lactate promotes M2-like macrophage polarization, although this response is concentration-dependent [195]. Thus, the role of lactate in tumors can be understood as ecological reprogramming that extends from metabolic adaptation to structural remodeling and immunosuppression.

### Lactylation in immune escape and therapy resistance

Under sustained lactate exposure, protein lactylation can convert a high-lactate microenvironment into more stable transcriptional and protein functional outputs. In tumors, lactylation has been linked to long-term immune escape, malignant adaptation, and therapy resistance. The translational relevance of these lactylation events therefore depends on whether defined lactylation sites establish functionally validated and therapeutically actionable dependencies, whereas an overall increase in lactylation alone is insufficient to establish target relevance.

Tumor-associated histone lactylation can promote immune escape and therapy resistance by altering transcriptional programs in both immune and tumor cells (Fig. 4c). In immune cells, tumor-derived lactate can induce H3K18la in naïve CD8⁺ T cells, activate nuclear receptor subfamily 4 group A member 1 (Nur77) transcription, and enhance tonic T-cell receptor (TCR) signaling, thereby rendering T cells hyporesponsive to antigen stimulation [101]. In gastric cancer, endoplasmic reticulum stress-induced H4K12la promotes protein disulfide-isomerase A6 (PDIA6) expression in dendritic cells, impairs dendritic-cell function, and reduces T-cell-mediated tumor killing, thereby facilitating immune escape [196]. In tumor cells, histone lactylation can reinforce both immune-checkpoint stability and resistance to cell death. In hepatocellular carcinoma, H3K18la-induced major vault protein (MVP) upregulation stabilizes PD-L1 by preventing β-transducin repeat-containing protein (β-TrCP)-mediated proteasomal degradation [197]. In lung adenocarcinoma, H4K8la-mediated transcriptional upregulation of aldo–keto reductase family 1 member C1 (AKR1C1) suppresses ferroptosis and maintains epidermal growth factor receptor tyrosine kinase inhibitor (EGFR-TKI) resistance [198]. These findings indicate that histone lactylation can promote immune escape through both immune-cell dysfunction and tumor-cell adaptation, while also supporting tumor-cell survival under therapeutic pressure.

Histone lactylation can also reinforce tumor adaptation through self-reinforcing metabolic transcriptional programs (Fig. 4c). In hepatocellular carcinoma, H4K5la is enriched at the ENO2 promoter and enhances ENO2 transcription, thereby promoting glycolysis and lactate production and establishing an H4K5la–ENO2 positive feedback loop [97]. In pancreatic ductal adenocarcinoma, glycolysis-induced H3K18la promotes the expression of TTK protein kinase (TTK) and BUB1 mitotic checkpoint serine/threonine kinase B (BUB1B), which further increase p300 expression, whereas TTK additionally phosphorylates and activates LDHA at Y239, thereby sustaining lactate production and H3K18la levels [199].

Non-histone lactylation provides a complementary mechanism of immune escape and therapy resistance by directly altering the stability, intracellular trafficking, and functional activity of key proteins (Fig. 4c). PD-L1 K280 lactylation reduces PD-L1 binding to HECT, UBA and WWE domain-containing E3 ubiquitin protein ligase 1 (HUWE1) and subsequent ubiquitination and proteasomal degradation [132], whereas PD-L1 K271 lactylation stabilizes PD-L1 by limiting its lysosomal degradation [120]. Non-histone lactylation can also enhance DNA repair and thereby sustain treatment resistance. MRE11 K673 lactylation increases the binding of MRE11 to DNA and promotes homologous recombination repair, thereby conferring resistance to cisplatin and poly(ADP-ribose) polymerase (PARP) inhibitors in tumor cells [119]. Other non-histone lactylation events, including those involving SPTAN1 [122], endosulfine alpha (ENSA) [130], and apolipoprotein C2 (APOC2) [134], can collectively contribute to immunosuppression and therapy resistance through pro-tumor signaling, immune-cell recruitment, and metabolic remodeling. Overall, histone lactylation generally explains persistent transcriptional bias, whereas non-histone lactylation often acts more directly by altering protein stability, localization, interactions, and activity. Together, these two regulatory layers provide a molecular basis for glycolytic dominance, immune escape, and therapy resistance.

## Therapeutic targeting of the lactate–lactylation axis

The central challenge in lactate-targeted therapy lies in mechanistic heterogeneity across lesions. The same high-lactate phenotype may be driven by enhanced lactate production, transport dependence, receptor-mediated sensing, or functional lactylation events, each of which corresponds to a distinct therapeutic window. Without sufficient mechanistic resolution, lactate-related therapy risks remaining at the level of broad metabolic suppression, making it difficult to predict which patients will benefit and potentially disrupting physiological lactate utilization and tissue repair. Therapeutic development should therefore not only reduce lactate pressure, but also identify the dominant lactate-axis dependency within a given lesion and select the corresponding intervention strategy accordingly.

### Targeting lactate production, transport, and sensing

**Fig. 5:**
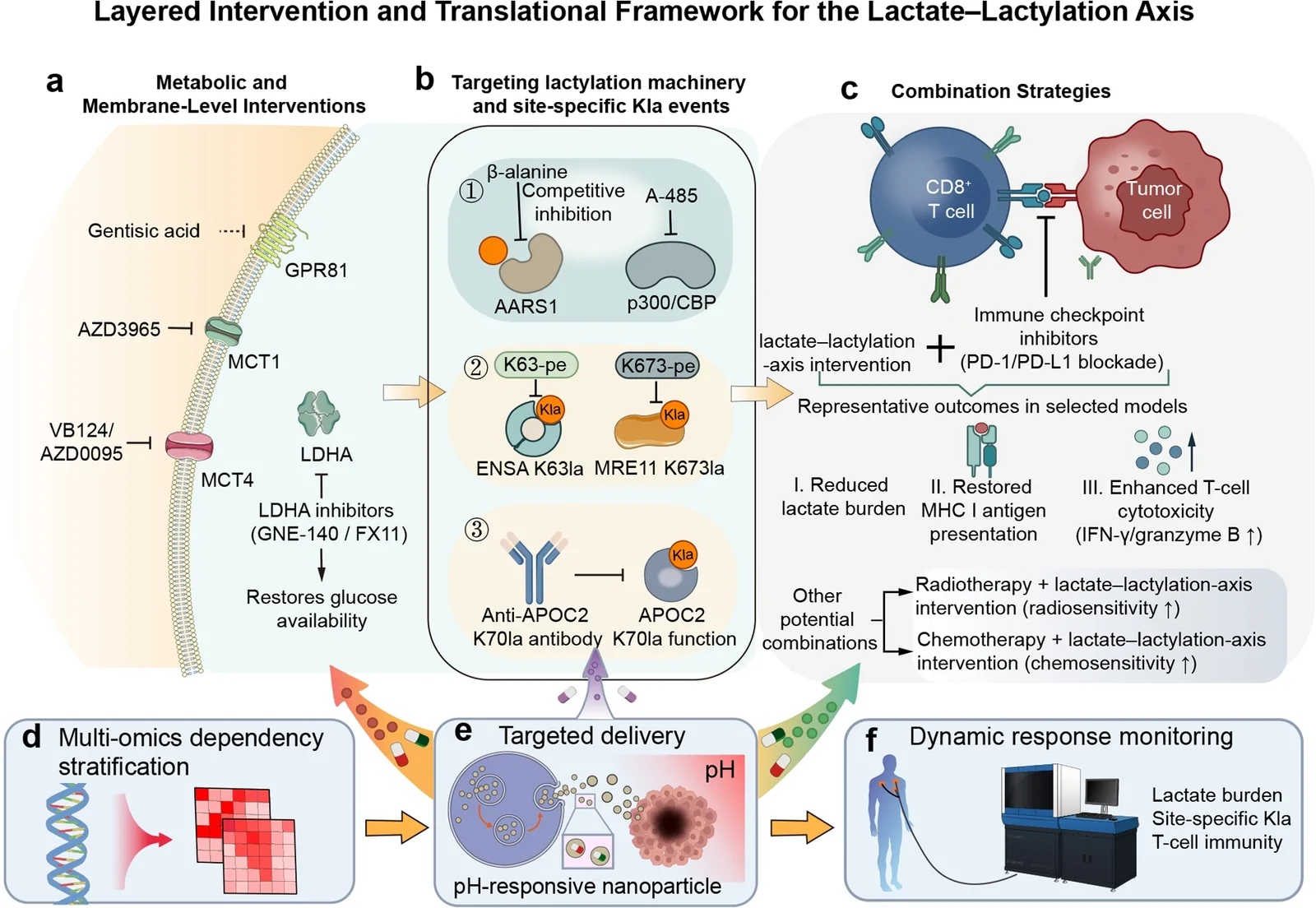
Layered intervention and translational framework for the lactate–lactylation axis. This schematic integrates therapeutic targeting of the lactate–lactylation axis with combination therapy and translational implementation. **a** Representative metabolic and membrane-level approaches inhibit lactate sensing through GPR81, lactate transport through MCT1 or MCT4, or lactate production through LDHA. **b** Lactylation-directed approaches include modulation of regulatory enzymes, competitive blockade of lactate binding to AARS1, site-blocking peptides targeting defined lactylation events, and antibodies directed against lactylated proteins. **c** Combination strategies pair lactate–lactylation-axis targeting with immune checkpoint blockade and may also incorporate radiotherapy or chemotherapy, with the aims of reducing lactate burden, restoring MHC I antigen presentation, and enhancing T-cell cytotoxicity or treatment sensitivity. **d**–**f** Multi-omics stratification, targeted delivery, and dynamic response monitoring support treatment selection and pharmacodynamic evaluation. Orange dots represent lactate; orange “Kla” symbols and the suffix “la” denote lysine lactylation at the indicated residue; ↑ and ↓ indicate increases and decreases, respectively

Research on lactate-targeted metabolism has gradually moved beyond conventional glycolytic inhibition toward relieving microenvironmental stress and enhancing immunometabolic sensitivity. LDH/LDHA inhibition represents a direct strategy for reducing lactate production. Early LDH inhibitors such as FX11 demonstrated the feasibility of suppressing glycolysis-dependent tumor growth [200]. LDH inhibition mediated by GNE-140 can reshape intratumoral glucose utilization, increase glucose availability for T cells, and enhance the efficacy of immune checkpoint blockade [201]. Targeting lactate transport is a more clearly advanced direction in clinical translation. The MCT1 inhibitor AZD3965 has completed a phase I dose-escalation study in patients with advanced solid tumors and lymphoma [202]. MCT4 inhibitors, including VB124, MSC-4381, and AZD0095, remain largely at the preclinical stage, but have shown potential to improve the immune microenvironment or enhance antitumor effects [203–205]. Lactate-sensing pathways also provide therapeutic entry points beyond inhibiting lactate production or blocking lactate transport. Early studies frequently used 3-OBA as a putative GPR81 antagonist, although its antagonistic activity against GPR81 has not been experimentally validated and it should not be regarded as a GPR81-selective probe [206]. Gentisic acid has more recently been characterized as a potential GPR81 inhibitor that suppresses lactate-induced EMT and mTOR signaling, prevents GPR81-mediated DEPDC5 degradation, and limits metastatic progression in colorectal cancer models [207]. These strategies correspond to three levels of lactate-axis intervention: production, transport, and sensing (Fig. 5a). The mechanistic positioning and reported combination-therapy status of these interventions are compared in Table 3.

**Table 3:** Representative therapeutic strategies targeting lactate metabolism, lactate sensing, and protein lactylation, with reported evidence or clinical status in combination with immune checkpoint inhibitors

Target	Representative agent	Mechanistic positioning	Reported combination evidence/status with ICIs
Lactate metabolism and sensing			
LDHA	FX11 (preclinical) [200]	Small-molecule LDHA inhibitor	Preclinical; combined with anti-PD-1 antibody upregulates MHC I and increases CD8⁺ T-cell infiltration [208]
	GNE-140 (preclinical) [209]	LDHA inhibitor	Preclinical; combined with anti-CTLA-4 antibody synergistically suppresses tumor growth and prolongs survival [201]
MCT1	AZD3965 (phase I completed; NCT01791595) [202]	MCT1 inhibitor	Preclinical; combined with PD-L1 antibody–drug conjugate overcomes lactate-mediated immunosuppression and resistance, restoring antitumor immune responses [210]
MCT1/MCT4	Syrosingopine (preclinical) [211]	Dual MCT1/MCT4 inhibitor	Preclinical; combined with anti-PD-1 therapy to suppress tumor growth, restore T-cell effector function, and prolong survival in immunocompetent triple-negative breast cancer models [212]
MCT4	VB124 (preclinical) [203]	MCT4 inhibitor	Preclinical; combined with anti-PD-1 therapy in HCC models, enhancing CD8⁺ T-cell infiltration and cytotoxicity and restoring PD-1-blockade sensitivity in ACVR2A-deficient, high-lactate tumors [213]
	MSC-4381 (preclinical) [204]	MCT4 inhibitor	Preclinical; combined with anti-PD-L1 antibody enhances antitumor immunity, increases T cell cytotoxicity and reduces exhaustion [204]
GPR81	Gentisic acid (preclinical) [207]	GPR81-associated blockade strategy	No direct combination evidence reported
Lactylation			
Lactylation	β-alanine (preclinical) [70]	Competitive blocker of lactate binding to AARS1	Preclinical; β-alanine synergizes with anti-PD-1 therapy in a GCMSC/PBMC tumor-bearing model, enhancing tumor regression and CD8⁺ T-cell effector function [95]
	A-485 (preclinical) [214]	p300/CBP catalytic inhibitor	Preclinical; combined with anti-PD-L1 antibody enhances antitumor effect, but available mechanistic evidence mainly points to acetylation-mediated PD-L1 regulation rather than a specific lactylation effect [215]
	K673-pe (preclinical) [119]	Site-blocking peptide targeting MRE11 K673 lactylation	No direct combination evidence reported
	K63-pe (preclinical) [130]	Site-blocking peptide targeting ENSA K63 lactylation	Preclinical; combination with anti-PD-1 therapy reverses treatment resistance and enhances antitumor efficacy in pancreatic ductal adenocarcinoma models [130]
	anti-APOC2 K70la antibody (preclinical) [134]	Antibody targeting lactylated APOC2	Preclinical; combined with anti-PD-1 antibody reverses treatment resistance and synergistically inhibits non-small-cell lung cancer growth [134]

### Targeting lactylation machinery and functional lactylation events

When a high-lactate state has already established stable transcriptional reprogramming or altered protein function, simply reducing lactate production or transport may be insufficient to reverse the existing pathological state. Therapeutic attention may need to extend toward functional lactylation events, particularly modifications that have been defined by site identification, validated effects on protein function, and demonstrated links to pathological phenotypes. Lactylation-targeted intervention can be developed around regulatory enzyme systems, functional modification sites, and downstream effector processes.

Enzyme systems such as p300, CREB-binding protein (CBP), AARS1/2, HDACs, and SIRTs provide pharmacological entry points for modulating lactylation [216]. The p300/CBP inhibitor A-485 and the AARS1 inhibitor β-alanine are commonly used to test the tractability of lactylation-targeted intervention. A-485 mainly acts on shared lysine acylation writer machinery and is therefore better suited as a tool for epigenetic modulation [214, 217]. β-alanine can competitively interfere with the binding of lactate to AARS1, reduce AARS1-mediated p53 lactylation, and suppress tumorigenesis in animal models [70]. However, because these enzymes regulate multiple forms of lysine acylation, direct translation into lactylation-specific therapy remains limited by selectivity.

Site-specific lactylation intervention is more closely aligned with precision therapy (Fig. 5b). MRE11 K673 lactylation enhances DNA binding and homologous recombination repair, thereby promoting resistance of tumor cells to cisplatin and PARP inhibitors. A cell-penetrating peptide targeting the K673 region can reduce MRE11 lactylation and restore drug sensitivity [119]. APOC2 K70 lactylation contributes to lipid metabolic remodeling by promoting free fatty acid (FFA) release, Treg accumulation, and immunotherapy resistance. An APOC2 K70-lactylation antibody provides preclinical support for antibody-based lactylation intervention [134]. Overall, global modulation of writers or erasers can be used to validate the pharmacological plasticity of lactylation, but insufficient specificity and off-target interference with other PTMs limit direct translation. Cell-penetrating peptides and site-specific antibodies are more closely linked to defined pathological nodes. Future studies need to improve modification specificity, delivery efficiency, and the ability to identify lesions that depend on lactylation.

### Combining lactate-axis interventions with immunotherapy and standard therapy

The most realistic clinical role of lactate-targeted intervention is not to replace existing therapies as a monotherapy, but to serve as a microenvironment-modulating and resistance-reversal module within combination regimens. In the context of immunotherapy, lactate accumulation, particularly when accompanied by extracellular acidification and glucose depletion, can restrict CD8⁺ T-cell glucose uptake and effector function, impair dendritic-cell antigen-presenting capacity, and favor the enrichment of myeloid-derived suppressor cells [189, 218]. Although immune checkpoint inhibitors (ICIs) can relieve inhibitory signaling mediated by programmed cell death protein 1 (PD-1)/PD-L1 or cytotoxic T-lymphocyte-associated protein 4 (CTLA-4), tumors with a high lactate burden may remain constrained by unfavorable T-cell metabolic conditions, impaired antigen presentation, and a suppressive myeloid milieu. Consistent with this model, cancer-cell-intrinsic lactate-associated programs have been linked to immune-cold tumor states and poorer outcomes in ICI-treated cohorts [219], whereas inhibition of tumor-cell MCT4 can restore CD8⁺ T-cell function and enhance anti-PD-1 efficacy in preclinical models [220]. The rationale for combining lactate-axis intervention with ICIs therefore lies in concurrently relieving metabolic immunosuppression and checkpoint-dependent immune inhibition.

Preclinical evidence already supports combining inhibitors of lactate production or transport with ICIs. Diclofenac-mediated inhibition of MCT1/MCT4-dependent lactate transport preserves T-cell effector function and enhances checkpoint therapy [221]. In small-cell lung cancer subtype A, LDHA-derived lactate suppresses MHC I antigen presentation through an H4K5la–PRC2 axis. Pharmacological LDHA inhibition with FX11 restores MHC I expression, and its combination with PD-1 blockade increases T-cell infiltration and synergistically suppresses tumor growth [208].

Blocking functional lactylation events may provide a more precise molecular entry point for ICI-based combinations. For example, a site-blocking peptide targeting ENSA K63 lactylation (K63-pe) reduces C–C motif chemokine ligand 2 (CCL2) secretion and immunosuppressive macrophage activity, and renders immune-excluded tumors more responsive to immunotherapy [130]. In a gastric cancer mesenchymal stromal-cell model, H4K8la promoted FAP transcription, ECM remodeling, and CD8⁺ T-cell exclusion, whereas β-alanine-mediated suppression of AARS1-associated lactylation enhanced the efficacy of anti-PD-1 therapy in vivo (Fig. 5c) [95].

Lactate-related interventions can also be incorporated into combinations with radiotherapy and chemotherapy. Lactate enrichment often coincides with hypoxia, extracellular acidification, metabolic adaptation, and treatment resistance. When combined with radiotherapy, inhibition of LDHA or MCT1 can disrupt lactate-dependent metabolic adaptation and reduce immunosuppression, thereby enhancing radiosensitivity and tumor control in preclinical models [222, 223]. When combined with chemotherapy, LDHA inhibition can disrupt glycolytic adaptation, increase apoptosis, and restore sensitivity to taxane-based treatment [224, 225].

Major translational limitations of combination therapy include systemic toxicity and the unresolved optimization of dose and treatment scheduling [202, 226]. LDH and MCTs participate in physiological lactate metabolism and metabolic homeostasis, whereas p300/CBP, HDACs, SIRTs, and AARS1/2 have broad functions in protein acylation, chromatin regulation, or canonical aminoacylation [70, 216]. This functional breadth raises the risk of on-target metabolic toxicity and collateral perturbation of non-lactylation-dependent processes; notably, dose-escalation of the MCT1 inhibitor AZD3965 produced primarily dose-dependent, reversible ocular toxicities, together with isolated cardiac troponin elevation and acidosis [202]. When ICIs are included, immune-related adverse events introduce an additional source of treatment toxicity [227]. Accordingly, lactate-axis combinations should initially be prioritized for biomarker-defined lesions with a dominant and pharmacologically reversible lactate-axis dependency, together with careful optimization of dose, treatment sequence, and regimen-specific toxicity.

### Patient stratification, lesion-selective delivery, and dynamic monitoring

Precision translation of therapies targeting lactate metabolism and lactylation requires simultaneous clarification of three issues: whether patients harbor an actionable lactate-axis dependency, whether the drug can act selectively on the lesion, and whether treatment induces the expected metabolic and immune remodeling. Circulating LDH levels and tumor MCT1/MCT4 expression can provide indirect evidence of a lactate-associated metabolic phenotype [228, 229], but these markers do not establish a specific therapeutic dependency or determine whether lactate has already generated a persistent pathological program through lactylation. Lactylomics, proteomics, and site-level functional validation can further identify lactylation events with potential causal relevance, such as MRE11 K673la [119] and PD-L1 K280 lactylation [132]. Single-cell and spatial multi-omics can further map whether lactate-associated metabolic states colocalize with immunosuppressive cellular ecosystems [4], whereas spatial validation of specific lactylation sites will require site-resolved protein-level measurements. This stratification framework can subdivide a broadly defined high-lactate state into therapeutically distinct scenarios characterized by dependence on lactate production, lactate transport, receptor-mediated sensing, or functional lactylation events (Fig. 5d).

Lesion-selective delivery aims to widen the therapeutic window while reducing interference with physiological lactate metabolism and normal lysine acylation programs. Acid- or lactate-responsive delivery systems can exploit tumor lactate enrichment and low-pH features to enable local release of lactate metabolism-modulating agents or biotherapeutic components [230, 231]. Co-delivery of lactate-degrading enzymes with immunomodulatory modules or gene-editing components may simultaneously reduce local lactate pressure and disrupt immune escape signaling [232]. For interventions targeting writers, erasers, or site-specific lactylation events, delivery systems should further improve cell-type selectivity so that therapeutic activity is preferentially directed toward tumor cells, suppressive myeloid cells, or defined stromal niches (Fig. 5e).

Dynamic feedback should cover metabolic input, lactylation signaling, and immune output (Fig. 5f). The metabolic input layer is used to assess changes in pyruvate-to-lactate conversion and LDH-associated metabolic flux, and can be assessed using approaches such as hyperpolarized ^13^C-pyruvate magnetic resonance imaging (MRI), chemical exchange saturation transfer (CEST)/acidity-based chemical exchange saturation transfer (acidoCEST) MRI, or electrochemical biosensors [233–237]. The lactylation signaling layer is used to confirm whether functional lactylation events have been attenuated [238]; this layer is better evaluated using paired pre- and post-treatment tissue samples through lactylation proteomics, targeted mass spectrometry, or immunological assays. The immune output layer is used to assess whether antitumor immunity has been restored. This assessment can integrate peripheral blood flow cytometry, cytometry by time of flight (CyTOF), cytokine profiling, or soluble immune checkpoint detection, together with multiplex immunofluorescence, co-detection by indexing (CODEX), or imaging mass cytometry (IMC) to determine whether local T-cell infiltration, myeloid-cell accumulation, and immune-exclusion patterns have improved [239–243]. Concordant serial changes across these layers would provide stronger evidence of target engagement and pharmacodynamic activity and would support the clinical evaluation of lactate-axis interventions.

Therefore, the translational pathway for therapies targeting lactate metabolism and lactylation should be centered on mechanistic dependency. After identifying a high-lactate state, the dominant lactate-axis component should be further validated, and therapeutic efficacy should be confirmed through selective intervention and dynamic monitoring. Defining the leading mechanism, selecting the corresponding intervention layer, and using serial readouts to confirm coordinated changes in metabolic pressure, lactylation signaling, and immune output are essential prerequisites for advancing lactate-related therapy into clinical validation.

## Conclusions and perspectives

Lactate biology has shifted from a descriptive marker of glycolytic stress to a mechanistically stratified field linking metabolic burden, microenvironmental remodeling, receptor-mediated signaling, and protein lactylation. Lactate can function as a reutilizable carbon source and a ligand for GPR81, while its accumulation coupled to proton export can contribute to extracellular acidification. Histone and non-histone lactylation can further translate persistent lactate exposure into transcriptional reprogramming and changes in protein stability, trafficking, molecular interactions, signaling, and immune function. The evidence supporting these conclusions should be interpreted hierarchically. Increased lactate, acidic pH, LDH/MCT upregulation, or pan-Kla signals define a lactate-stressed background. Site-specific Kla identifies candidate modification events, whereas functional regulatory events require evidence that the modification alters protein behavior or cellular phenotype. Disease dependency and therapeutic relevance further require genetic or pharmacological perturbation showing reversal of the pathological phenotype.

Recent tumor studies further emphasize source, niche, and substrate specificity. In glioblastoma (GBM), TAM-derived lactate enters glioma stem cells (GSCs) through the MCT4–MCT1 axis, induces KU70 K317 lactylation, suppresses cGAS–STING signaling, and contributes to immune escape and therapy resistance [131]. In KRAS-mutant colorectal cancer, extracellular lactate promotes CD44 lactylation on CD8⁺ T cells and weakens hyaluronan binding and AKT signaling [133]. In lung adenocarcinoma, FAP⁺ CAFs create a long intergenic non-protein-coding RNA 1711 (LINC01711)–fibroblast growth factor receptor 1 (FGFR1)–LDHA/MCT4-dependent lactate barrier that limits CD8⁺ T-cell infiltration and reduces anti-PD-1 efficacy [244]. In NK cells, lactate-driven Kla disrupts NAD⁺ metabolism and Rho-associated coiled-coil-containing protein kinase 1 (ROCK1)–dynamin-related protein 1 (DRP1)-associated mitochondrial homeostasis, reducing cytotoxicity [245]. These findings support a shift from treating lactate as a general suppressive metabolite to mapping discrete lactate–lactylation circuits within defined cellular niches.

Clinical translation should follow the same layered logic. Patients first need to be stratified according to the dominant lesion dependency, including lactate production, lactate transport, receptor sensing, or functional lactylation events. LDH/LDHA, MCT1/MCT4, and GPR81 targeting mainly aims to reduce lactate pressure and improve the local immune-metabolic environment [246–248]. In contrast, writer or eraser modulation remains better suited to mechanism validation and target discovery at the current stage, because these enzymes regulate multiple lysine acylation programs and lack sufficient lactylation specificity. Greater therapeutic precision is more likely to come from strategies directed at defined Kla sites or lactylation-dependent pathological pathways.

A major barrier to clinical translation is the difficulty of continuously and mechanistically monitoring lactate-axis activity within individual lesions. Lactate and pH measurements are closest to the metabolic-input layer, but current metabolic imaging and invasive sampling approaches remain constrained by spatial resolution, tissue accessibility, calibration requirements, and intralesional heterogeneity. Emerging microfluidic and genetically encoded lactate sensors enable real-time or high-resolution lactate measurements in experimental systems, although their applicability to longitudinal monitoring of clinical tumor lesions remains to be established [236, 249]. Current site-resolved lactylation analysis relies largely on sampled tissues, mass spectrometry, and validated site-specific antibodies, making repeated assessment of dynamic Kla changes within the same lesion difficult [56, 78]. Immune-output readouts, including peripheral immune profiling, cytokine and soluble-checkpoint measurements, CyTOF, SERS-based multiplex assays, and spatial immune imaging, can evaluate treatment-associated immune changes but cannot by themselves establish whether lactate pressure or functional lactylation has been suppressed [239, 240, 250, 251]. These limitations indicate that no single readout is sufficient for lactate-axis-guided therapy.

Future monitoring systems should integrate metabolic input, lactylation mechanisms, and immune output across serial assessments of the same lesion. Metabolic imaging or validated local sensors could determine whether lactate-associated metabolic pressure decreases, whereas paired tissue analysis using lactylomics, targeted mass spectrometry, spatial proteomics, or site-specific imaging assays could test whether the targeted functional lactylation events are attenuated. Local and peripheral immune profiling could then determine whether antitumor immunity recovers in parallel. Beyond suppressing lactate pressure, emerging strategies may also exploit the lactic-acid-rich and acidic tumor microenvironment, as illustrated by lactic acid-responsive promoter-based chimeric antigen receptor (CAR) T cells that use this metabolic feature to drive lesion-selective CAR expression [252]. Future work should prioritize lesion-level dependency identification, site-level functional validation, selective delivery, and longitudinal pharmacodynamic readouts, allowing lactate–lactylation research to connect mechanism, target selection, and precision therapy more directly.

## Data Availability

Not applicable.

## Abbreviations

AARS1/2: Alanyl-tRNA synthetase 1/2
acidoCEST: Acidity-based chemical exchange saturation transfer
ACSS2: Acetyl-CoA synthetase 2
ACVR2A: Activin A receptor type 2A
AK2: Adenylate kinase 2
AKR1C1: Aldo-keto reductase family 1 member C1
AKT: Protein kinase B
APACHE II: Acute Physiology and Chronic Health Evaluation II
APOC2: Apolipoprotein C2
ASIC1a/3: Acid-sensing ion channels 1a/3
ASC: Apoptosis-associated speck-like protein containing a caspase recruitment domain
ATP: Adenosine triphosphate
BCG: Bacillus Calmette–Guérin
BMDMs: Bone marrow-derived macrophages
BMP7: Bone morphogenetic protein 7
BUB1B: BUB1 mitotic checkpoint serine/threonine kinase B
CAFs: Cancer-associated fibroblasts
cAMP: Cyclic adenosine monophosphate
CAR: Chimeric antigen receptor
CBFB: Core-binding factor subunit beta
CBP: CREB-binding protein
CCL2: C-C motif chemokine ligand 2
CCSCs: Colorectal cancer stem cells
CD4: Cluster of differentiation 4
CD44: Cluster of differentiation 44
CD8: Cluster of differentiation 8
CEST: Chemical exchange saturation transfer
cGAMP: Cyclic GMP–AMP
cGAS: Cyclic GMP–AMP synthase
CHIP: C terminus of Hsc70-interacting protein
CIITA: Class II major histocompatibility complex transactivator
CoA: Coenzyme A
CODEX: Co-detection by indexing
CTLA-4: Cytotoxic T-lymphocyte-associated protein 4
CyTOF: Cytometry by time of flight
DAS28: Disease Activity Score in 28 joints
DC: Dendritic cell
DRP1: Dynamin-related protein 1
ECM: Extracellular matrix
EGFR-TKI: Epidermal growth factor receptor tyrosine kinase inhibitor
ENO2: Enolase 2
ENSA: Endosulfine alpha
FAP: Fibroblast activation protein
FFA: Free fatty acid
FGFR1: Fibroblast growth factor receptor 1
GBM: Glioblastoma
GCMSCs: Gastric cancer mesenchymal stromal cells
GCN5: General control nonderepressible 5
GPR132: G protein-coupled receptor 132
GPR81: G protein-coupled receptor 81
GPX4: Glutathione peroxidase 4
GSC: Glioma stem cell
H3K18la: Histone H3 lysine 18 lactylation
H3K27ac: Acetylation of histone H3 lysine 27
H3K4me3: Trimethylation of histone H3 lysine 4
HBV: Hepatitis B virus
HCC: Hepatocellular carcinoma
HDAC: Histone deacetylase
HES1: HES family bHLH transcription factor 1
HIF-1: Hypoxia-inducible factor 1
HIF-1α: Hypoxia-inducible factor 1α
HMGB1: High mobility group box 1
HR: Homologous recombination
HSPA12B: Heat shock protein family A member 12B
HUWE1: HECT, UBA and WWE domain-containing E3 ubiquitin protein ligase 1
ICIs: Immune checkpoint inhibitors
IFN-I: Type I interferon
IFN-γ: Interferon-γ
IL: Interleukin
IMC: Imaging mass cytometry
JAK2: Janus kinase 2
Kla: Lysine lactylation
KLF4: Krüppel-like factor 4
KRAS: KRAS proto-oncogene, GTPase
LAMP1: Lysosome-associated membrane glycoprotein 1
LC–MS/MS: Liquid chromatography–tandem mass spectrometry
LC3: Microtubule-associated protein 1 light chain 3
LDH: Lactate dehydrogenase
LDHA: Lactate dehydrogenase A
LINC01711: Long intergenic non-protein coding RNA 1711
LPS: Lipopolysaccharide
MAVS: Mitochondrial antiviral-signaling protein
MCT 1/4: Monocarboxylate transporter1/4
METTL1: Methyltransferase 1
MHC: Major histocompatibility complex
MICU3: Mitochondrial calcium uptake 3
MRE11: MRE11 homolog, double-strand break repair nuclease
MRI: Magnetic resonance imaging
MRN: MRE11–RAD50–NBS1 complex
mTOR: Mechanistic target of rapamycin
MVP: Major vault protein
MYC: MYC proto-oncogene, bHLH transcription factor
NAD⁺: Oxidized nicotinamide adenine dinucleotide
NADH: Reduced nicotinamide adenine dinucleotide
NBS1: Nijmegen breakage syndrome protein 1
NET: Neutrophil extracellular trap
NeuroD1: Neuronal differentiation 1
NF-κB: Nuclear factor-κB
NK: Natural killer
NLRP3: NOD-, LRR-, and pyrin domain-containing protein 3
NOTCH1: Notch receptor 1
Nur77: Nuclear receptor subfamily 4 group A member 1
Olig2: Oligodendrocyte transcription factor 2
p300: E1A-binding protein p300
PARP: Poly(ADP-ribose) polymerase
PBMCs: Peripheral blood mononuclear cells
PD-1: Programmed cell death protein 1
PD-L1: Programmed death-ligand 1
PDIA6: Protein disulfide-isomerase A6
PFKP: Phosphofructokinase, platelet type
PI3K: Phosphoinositide 3-kinase
PKM2: Pyruvate kinase M2
PKR: Protein kinase R
PP2A: Protein phosphatase 2A
PRC2: Polycomb repressive complex 2
PTM: Post-translational modification
RA-FLS: Rheumatoid arthritis fibroblast-like synoviocyte
Rab7b: Ras-related protein Rab-7b
Rab11: Ras-related protein Rab-11
RelA: RELA proto-oncogene, NF-κB subunit
RLRs: Retinoic acid-inducible gene I-like receptors
ROCK1: Rho-associated coiled-coil-containing protein kinase 1
RUBCN: Rubicon autophagy regulator
S100A9: S100 calcium-binding protein A9
SERS: Surface-enhanced Raman scattering
SIRT: Sirtuin
SOFA: Sequential Organ Failure Assessment
SPTAN1: Spectrin alpha, non-erythrocytic 1
SREBP2: Sterol regulatory element-binding protein 2
STAT1: Signal transducer and activator of transcription 1
STING: Stimulator of interferon genes
TAM: Tumor-associated macrophage
TCA: Tricarboxylic acid cycle
TCR: T-cell receptor
TFEB: Transcription factor EB
TGF-β1: Transforming growth factor-β1
Th17: T helper 17 cell
TME: Tumor microenvironment
TNF-α: Tumor necrosis factor-α
Treg: Regulatory T cell
TTK: TTK protein kinase
VDAC1: Voltage-dependent anion channel 1
VHL: Von Hippel–Lindau
β-TrCP: β-Transducin repeat-containing protein
